# Carbon-Based Nanomaterials for Biomedical Applications: A Recent Study

**DOI:** 10.3389/fphar.2018.01401

**Published:** 2019-03-11

**Authors:** Debabrata Maiti, Xiangmin Tong, Xiaozhou Mou, Kai Yang

**Affiliations:** ^1^State Key Laboratory of Radiation Medicine and Protection, School of Radiation Medicine and Protection, School for Radiological and Interdisciplinary Sciences (RAD-X), Collaborative Innovation Center of Radiation Medicine of Jiangsu Higher Education Institutions, Soochow University, Suzhou, China; ^2^Key Laboratory of Tumor Molecular Diagnosis and Individualized Medicine of Zhejiang Province, Zhejiang Provincial People’s Hospital, Hangzhou, China

**Keywords:** carbon nanomaterials, biomedical applications, biosensor, drug delivery, cancer therapy

## Abstract

The study of carbon-based nanomaterials (CBNs) for biomedical applications has attracted great attention due to their unique chemical and physical properties including thermal, mechanical, electrical, optical and structural diversity. With the help of these intrinsic properties, CBNs, including carbon nanotubes (CNT), graphene oxide (GO), and graphene quantum dots (GQDs), have been extensively investigated in biomedical applications. This review summarizes the most recent studies in developing of CBNs for various biomedical applications including bio-sensing, drug delivery and cancer therapy.

## Introduction

**FIGURE 1:**
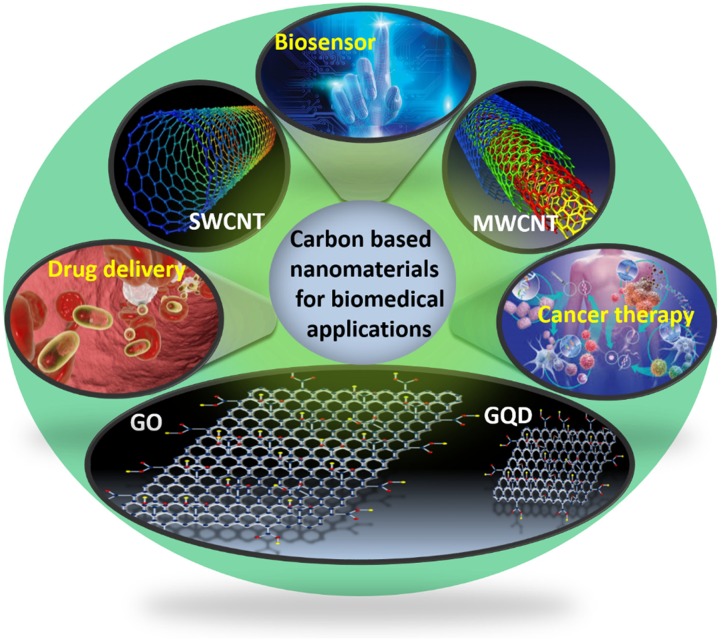
Schematic illustration for the biomedical applications of carbon-based nanomaterials (CBNs).

In the field of science and technology, carbon-based nanomaterials (CBNs) are becoming attractive nanomaterials (Cha et al., 2013; Wang et al., 2014, 2015; Tiwari et al., 2015; Lin et al., 2016; Mukhopadhyay et al., 2016; Zhang et al., 2017). Due to the existence of diverse allotropes of carbon, from renowned allotropic phases such as amorphous carbon, graphite and diamonds to newly discovered auspicious carbon nanotubes (CNTs), graphene oxide (GO), graphene quantum dots (GQDs) and fullerene, carbon-based materials have recently become prized (Mostofizadeh et al., 2011). Each member of the carbon family exhibits inimitable features and has been widely exploited in diverse biological applications including biosensing, drug delivery, tissue engineering, imaging, diagnosis and cancer therapy (Hong et al., 2015; Bhattacharya et al., 2016). In 1991, Sumio Iijima first observed the formation of multiwall CNTs from carbon arc discharge. After some years, Prof. Sumio Iijima and Donald Bethune individually perceived single wall CNTs (Monthioux and Kuznetsov, 2006). Afterward, research on CNTs proliferated quickly. CNTs were described as hollow cylinders consisting of graphitic sheets and were classified into single walled carbon nanotube (SWCNT) and multi walled carbon nanotube (MWCNT) (Figure 1). SWCNTs, with a cylindrical nanostructure, are made by rolling up a single graphitic sheet with a high aspect ratio. MWCNTs contain few graphitic layers in the rolling pattern, with an interlayer spacing of 3.4Å (Odom et al., 1998; Eatemadi et al., 2014). As a consequence of its unique mechanical, electrical and structural diversity, it gives superior strength, flexibility and electrical conductivity toward various biological entities, which is useful for sensing, medical diagnosis and treating various diseases (Wu et al., 2010; Hwang et al., 2013; Roldo and Fatouros, 2013; Kumar et al., 2017). However, among the various allotropes of carbon, graphene is considered the most attractive material owing to its unique intrinsic properties. About 70 years ago, in 1947, Wallace evaluated the electronic structure of graphene and McClure deduced the corresponding wave equation in 1956. The name “graphene” was first introduced in 1987 by Mouras and co-workers as “graphitic intercalation compounds (GIC)” (Sun et al., 2013). Over the last two decades, research on graphene has greatly increased, and various exceptional properties have been observed by investigators. Graphene is described as the planar graphitic sheet of graphite, consisting of sp^2^ hybridized carbon network with a carbon-carbon distance of 1.42Å and an interlayer spacing of 3.4Å (Figure 1; Erickson et al., 2010). Graphene exhibits a number of exceptional properties that lend to its potential favorability for bio-applications. The prospect of easy functionalization causes the enrichment of functional groups on its surface, which in turn facilitates the specific and selective detection of several biological segments. Furthermore, its extremely large surface area, chemical purity and free π electrons render it an ideal candidate for drug delivery (Yang et al., 2013; Zhang et al., 2013; Pattnaik et al., 2016). Moreover, with the help of its feasible behavior toward different fluorescent dyes, therapeutic agents and other biomaterials, it is widely used for *in vivo* imaging, diagnosis and treatment of cancer. Another recently invented and attractive biomaterial from the carbon family is GQDs, which is defined as a zero-dimensional graphene sheet with a lateral dimension of less than 100 nm in one to a few layers (3–10) (Song et al., 2015). During the conversion of two-dimensional graphene sheets into GQDs, the GQDs endow excellent photoluminescence due to quantum confinement (Wang et al., 2016). Interestingly, as compared to other fluorescent dye or semiconductor quantum dots, the GQDs exhibit superior biocompatibility and resistance to photo-bleaching. Additionally, GQDs carry keen features of graphene, such as a large surface area and available π electrons, which make the GQDs a smart nanomaterial for a wide range of biomedical applications, including imaging, targeted drug delivery, biomolecules sensing, cancer therapy and so on (Zheng et al., 2015; Kumawat et al., 2017; Li et al., 2017; Chen et al., 2018).

Recently, by utilizing the inherent properties of different newly invented CBNs, these have been modified and extensively used in biomedicine, including applications for bio-sensing, drug delivery and cancer therapy. This encouraged us to conduct a comprehensive review on CBNs in biomedical applications. Regarding the same issue, a few more reviews and prospective articles have been conducted, and most of them have discussed synthesis, characterizations and, to a lesser extent, biomedical applications. Moreover, many of these review articles have discussed overall research that has been carried out over last two decades. In this review we thoroughly recapitulate the most recent progress of CBNs for biomedical applications in the last half decade and offer our own point of view of the field. We expect that this review article will direct researchers to design developed CBNs for superior biomedical applications.

## Carbon Nanotubes (CNTs) for Biomedical Applications

### Carbon Nanotubes as Biosensors

Owing to their exceptional structural, mechanical, electronic and optical properties, CNTs have been regarded as a new generation nanoprobes (Tîlmaciu and Morris, 2015). Their high aspect ratio, high conductivity, high chemical stability and sensitivity (Zhao et al., 2002) and fast electron-transfer rate (Lin et al., 2004) make them exceedingly fit for biosensing applications. The basic element of CNT-based biosensors is the immobilization of biomolecules on its surface, therefore enhancing recognition and the signal transduction process. On the basis of their target recognition and transduction mechanisms, these biosensors are largely categorized into electrochemical and electronic CNT-based biosensors and optical biosensors. CNTs have been renowned as promising materials for improving electron transfer, which makes them appropriate for combining electrochemical and electronic biosensors (Jacobs et al., 2010; Holzinger et al., 2014; Kumar et al., 2015; Wang et al., 2015; Yang et al., 2015; Hou et al., 2016; Zribi et al., 2016).

**FIGURE 2:**
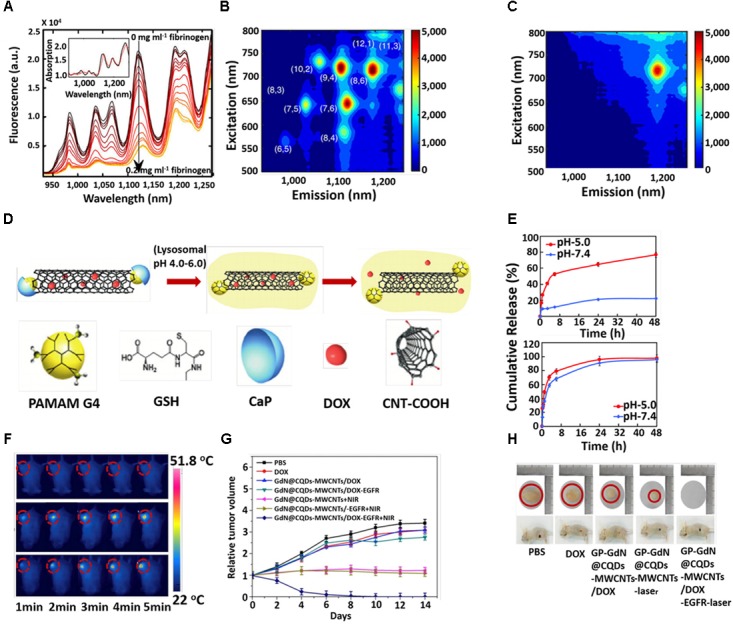
**(A)** Fluorescence spectra of 1,2-bis(diphenylphosphino) ethane (DPPE)-polyethylene glycol (PEG)-SWCNT with different concentrations of fibrinogen. Excitation-emission of DPPE-PEG-SWCNT solution **(B)** before and after **(C)** fibrinogen adding. **(D)** Schematic illustration for the triggered release of DOX from DOX-loaded CaP nanocapsule under intracellular endo/lysosomal conditions. **(E)** DOX release profile at pH 7.4 and 5 with time from CNT–G4–GSH–CaP–DOX and (upper) CNT–G4–GSH–DOX (lower). **(F)** *In vivo* photothermal images under 5 min NIR laser (808 nm, 1 W/cm^2^) irradiation. **(G)** Tumor growth curves after different treatments at different times. **(H)** Digital photographs of tumors and tumor-bearing mice after different treatments. Copyright Bisker et al. (2016) Nature publishing group, Banerjee et al. (2015) Royal Society of Chemistry, and Zhang et al. (2017) Elsevier.

Numerous CNT-glucose biosensors based on the conjugation of glucose oxidase have been designed. Zhu et al. (2014) used carbon nanotube non-woven fabrics (CNTFs) to sense glucose from a glucose oxidase-impregnated polyvinyl alcohol solution. The Gaitan Group have emphasized the effect of surface chemistry and the structure of glucose oxidase-coated MWCNT in electrochemical glucose sensing (Gaitán et al., 2017). Electrochemical biosensors built on CNTs have further been designed for detecting nitric oxide and sensing epinephrine (Ulissi et al., 2014; Mphuthi et al., 2016). Bisker et al. (2016) established 20 distinct SWCNT corona phases for detecting human blood proteins. The study revealed that the specific corona phase was capable of recognizing fibrinogen with high selectivity and resulted in a decrease of florescence intensity of SWCNT >80% at saturation (Figure 2A). However, absorption intensity remained unchanged with little red shift (Figure 2A, inset). The fluorescent response of SWCNT with a smaller diameter was more pronounced compared to the larger diameter nanotube, displayed in the excitation–emission profiles of the SWCNT sample before (Figure 2B) and after (Figure 2C) the fibrinogen adding. The fibrinogen recognition was tested in the human blood serum environment. Recently, the same group demonstrated that label-free detection of individual proteins’ efflux from *Escherichia coli* (bacteria) and *Pichia pastoris* (yeast) in real time was possible by using SWCNT (Landry et al., 2017). Baldo et al. (2016) successfully developed a MWCNT-based device detecting arginase-1. The Tuan Group developed a CNT-based field effect transistor (FET) as a conducting channel with a length and width of 15 and 700 μm. The CNT-based field effect transistor (CNTFET) was used directly in a DNA solution under a high current of 1.91 A (Xuan et al., 2017). The Zhou Group has explored the DNA-mediated SERS property of SWNTs, which permitted the ultrasensitive detection of a broad range of ctDNA in human blood. The T-rich de-oxy-ribonucleic acid (DNA)-mediated surface-enhanced Raman scattering (SERS) of SWNTs could sense a KRAS G12DM content as low as 0.3 fM, with a detection of 5.0 μL from the sample volume (Zhou et al., 2016). Their photophysical properties, such as fluorescence emission in the NIR region and excellent photo stability, make SWCNTs effective optical probes in biomedicine. Jena et al. (2017) designed single-stranded DNA functionalized SWCNTs, which responded to the lipid content in the endosomal lumen of live cells. From NIR photoluminescence of the SWCNTs, the lipid content was measured via solvatochromic shift (Jena et al., 2017).

### Carbon Nanotubes for Drug Delivery

Among the different carbon allotropes, CNTs have attracted escalating attention as a highly competent vehicle for transporting various drug molecules into the living cells because their natural morphology facilitates non-invasive penetration across the biological membranes (Chen et al., 2008; Das et al., 2013; Liu et al., 2013; Panczyk et al., 2016). Generally, drug molecules are attached to CNT sidewalls via covalent or non-covalent bonding between the drug molecules and functionalized CNT. But each of these processes has advantages or disadvantages. The covalent interaction makes the drug-loaded CNT stable in both the extra- and intracellular compartments, meaning that such a phenomenon has a lack of sustained release of the drug inside the cellular microenvironment of cancer cells, which is a shortcoming in the drug delivery system. Non-covalent interaction facilitates the controlled release of the drug in the acidic condition of tumor sites but suffers from stability in extracellular pH levels. Hence, the utilization of the inner hollow cavity of CNT for drug loading provides the ideal isolation of the drug from the physiological environment. In order to overcome the discrepancy of drug release in the tumor cell microenvironment, some external stimuli have been tested via temperature, electric field, light or a combination of these. To evaluate the temperature-responsive release of biomolecules, the Shin Group fabricated chitosan-functionalized CNT with thermosensitive polymer, poly-N-Isopropyl acryl amide (NIPAAm) and 1-butyl-3-. 21 vinyl imidazolium (NIPAAm-co-BVIm), followed by encapsulating the bovine serum albumin (BSA) at body temperature (37°C). The release of the BSA occurred just above the lower critical solution temperature (LCST) of poly-VBIm (38–40°C) (Kang et al., 2017). Shi et al. (2015) used an electric field to release the ibuprofen from a hybrid hydrogel composed of sodium alginate (SA), bacterial cellulose (BC), and multi-walled carbon nanotubes (MWCNTs). Estrada et al. (2013) studied the temperature and near infrared (NIR) light-responsive release of methylene blue (MB) from multi-walled carbon nanotube (MWCNT)–k-carrageenan hydrogel. However, to date, many drugs have been loaded onto the CNT including doxorubicin (Huang et al., 2011), paclitaxel (Singh et al., 2016), docetaxel (Raza et al., 2016), oxaliplatin (Lee et al., 2016), etc., to demonstrate the efficiency for *in vitro* and *in vivo* cancer treatments. The Dai Group have extensively studied functionalized CNT for the purpose of *in vitro* and *in vivo* drug delivery (Dhar et al., 2008; Liu et al., 2008, 2009a,b). Their group discovered a new strategy to make CNT highly water soluble to entrap drug molecules (Liu et al., 2007). The Jain Group evaluated and compared the *in vitro* and *in vivo* cancer targeting tendency of doxorubicin (DOX)-loaded folic acid (FA) and estrone (ES)-anchored PEG functionalized MWCNTs (DOX/ES-PEG-MWCNTs) on MCF-7 tumor-bearing Balb/c mice (Mehra and Jain, 2015). After 43 days, the mice treated with DOX/ES-PEG-MWCNTs showed a longer survival span compared to those groups treated with free DOX (18 days) or PBS (12 days). The Khandare Group reported calcium phosphate (CaP)-crowned drug loaded multiwall carbon nanotubes (CNT–GSH–G4–CaP) could be considered as a nanocapsule for intracellular delivery of an anticancer drug (Banerjee et al., 2015). The schematic diagram for the encapsulation and release of drug molecules from the nanocapsule is described in Figure 2D. They systematically studied pH triggered CaP dissolution and drug release in subcellular compartments such as lysosomes (pH5.0) (Figure 2E). Additionally, zero premature release at physiological pH supported the drug-loaded nanocapsule for effective anticancer treatment. Risi et al. (2014) steadily observed the efficient loading and releasing of a new anticancer drug on CNT. In order to improve the biocompatible nature of CNT, Xu et al. (2016) developed an amine-terminated PEG functionalized polydopamine (PDA) (shell)-CNT (core) nanosystem for drug delivery. The Picaud group investigated theoretically on the loading and releasing of cisplatin onto/from CNT (Mejri et al., 2015).

### Carbon Nanotubes for Cancer Therapy

Carbon nanotubes are widely used in biomedical applications due to their versatile properties. These are the attractive candidates for the carrying of anticancer drugs, genes and proteins for chemotherapy (Adeli et al., 2013; Eskandari et al., 2014; Amenta and Aschberger, 2015; Hwang et al., 2017). Moreover, strong NIR light absorption capability renders CNTs as efficient photothermal agents. Su et al. (2017) developed iRGD-polyethyleneimine (PEI) functionalized MWCNT followed by conjugation with candesartan (CD). The functionalized iRGD-PEI-MWCNT-CD was assembled with plasmid AT (2) [pAT (2)]. iRGD and CD were used to target αvβ3-integrin and AT1R of tumor endothelium and lung cancer cells, respectively. The CD as a chemotherapeutic exhibited synergistic downregulation of VEGF upon combining with pAT (2) and inhabited angiogenesis effectively (Su et al., 2017). The Zhou group designed a DOX-loaded MWCNT-magneto fluorescent carbon quantum dot (CQD) nanocomposite for chemo- and photothermal therapy (Zhang et al., 2017). The negative surface charge of the GdN@CQDs-MWCNTs facilitated binding with positively charged DOX molecules. The material had a high ability to absorb NIR light. On *in vivo* photothermal therapy, the temperature of the tumor site of the mice treated with GdN@CQDs-MWCNTs/DOX-EGFR was increased to 51.8°C under laser irradiation at the power density at 2 W/cm^2^ for 5 min. No significant change in temperature of the control group treated the mice’s tumor site (Figure 2F). This heating effect favored the release of DOX and photothermal therapy, as revealed by the suppression of tumor volume (Figures 2G,H). Recently, Dong et al. (2017) used DOX-loaded TAT-chitosan functionalized MWCNT nanosystem for combining chemo and photothermal therapy. In order to enhance apoptosis in cancer cells, the Dong-woo group used a PEG-coated CNT-ABT737 nanodrug to target mitochondria (Kim et al., 2017). Cytosol release of the nanondrug resulted in apoptosis of lung cancer cells through abruption of the mitochondrial membrane. Finally, the material exhibited effective *in vivo* therapeutic efficacy. Moreover, the localized heating effect under NIR irradiation induced therapeutic performance. The Chen Group developed a gold nanoparticle-coated carbon nanotube ring (CNTR) with superior Raman and optical signal properties, resulting in the improvement of the photoacoustic (PA) signal and photothermal conversion behavior of the CNTR@Au (Song et al., 2016). The material exhibited a significant outcome in image-guided cancer therapy. The surface plasmon resonance (SPR) absorption by gold in SWNT-Au-PEG-FA nanomaterials improved photothermal cancer killing efficacy (Wang et al., 2012; Bao et al., 2016). Some current observations based on CNTs for different cancer therapy have listed in Table 1.

**Table 1:** Use of different carbon-based nanomaterials for various cancer therapy.

Carbon-based nanomaterials	*In vitro*	Therapy	Reference
NY-ESO-1, CpG-ODNs with MWCNT	Dendritic cells	Immunoresponse	Faria et al., 2014
Magnetic ferrite nanoparticles filled	CNTSKOV3 cells	Imaging and therapy	Liu et al., 2014
PEG functionalized MWCNTS	U87, U373MG, NHA	Brain tumor therapy	Eldridge et al., 2016
CNT	-	Microbeam radiation therapy	Zhang et al., 2014
CNT	-	Microbeam radiation therapy	Hadsell et al., 2014
MWCNT	PANC-1	Pancreatic cancer	Mocan et al., 2014
MWCNT	HeLa	Photothermal therapy	Sobhani et al., 2017
SWCNT	4T1	Chemo-photothermal therapy	Yang et al., 2018
SWCNT	4T1	Photothermal	Liang et al., 2014
Porphyrin immobilized NanoGO	U87MG, HBMEC, ACBRI376	Photothermal	Su et al., 2016
Nano graphene sheet	-	Photothermal	Yang et al., 2010
Double network structured GO	HCT116	Chemo-photothermal	Fiorica et al., 2017
(PEG-g-PDMA-HA)@rGO	MDAMB-231, A549	Photothermal	Kim et al., 2015
GO decorated Ru(II)-PEG complex	A549	Photodynamic-photothermal	Zhang et al., 2017
Iron oxide-GO	HeLa	Chemo-photothermal	Deng et al., 2016
GO (^188^Re)-modified Fe_3_O_4_/silica	-	Chemo-photothermal	Yang et al., 2017
(HA)-modified Q-Graphene	A549, MRC-5	Chemotherapy	Luo et al., 2016
CuS-GO	HeLa	Chemo-photothermal	Han et al., 2017
RGO-PEG	U87	Chemo-photothermal photodynamic	Liu et al., 2017
GQD-Ce6-HA	A549	Photodynamic	Nafiujjaman et al., 2016
UCNP-GQD	4T1	Photodynamic	Zhang et al., 2017
7Gd-encapsulated Graphene Carbon	SSC-7	Photodynamic	Chen et al., 2018
GQDs	BT-474, MCF-7	-	Ko et al., 2017
GQDs	PANC-1, A-549, HepG2	-	Fan et al., 2017
GQDs	RG2	Chemotherapy	Su et al., 2017
GQDs	La29, HaCaT, Mia-Pa-Ca-2	Photothermal photodynamic	Thakur et al., 2017
GQDs	SW620, HCT116	Radiotherapy	Ruan et al., 2018


## Graphene Oxide for Biomedical Applications

### Graphene Oxide as Biosensor

**FIGURE 3:**
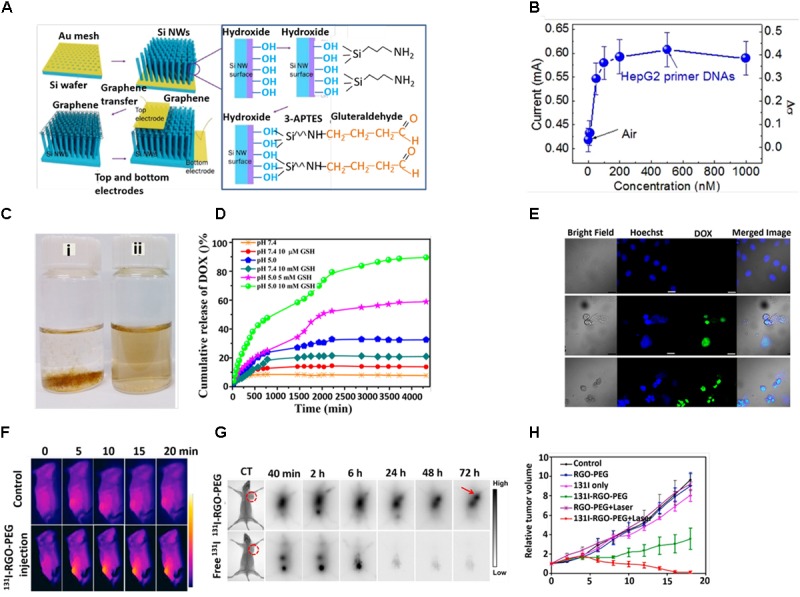
**(A)** Schematic diagram of fabrication process. **(B)** Responsivity of graphene/Si-NWs biosensors: Current of a graphene/Si-NWs biosensor as a function of mole fraction of p-ss oligonucleotide. **(C)** Digital photographs of aqueous dispersion of (i) GON and (ii) GON-Cy-ALG-PEG in PBS. **(D)** DOX release profile at pH 7.4 and 5 in presence and absence of GSH. **(E)** Cellular uptake of HepG2 cells stained by Hoechst (blue), DOX (green)-loaded GON-Cy-ALG-PEG in presence and absence of GSH. Bars represent 30 μm. **(F)** *In vivo* photothermal therapy **(G)** *in vivo* gamma imaging study. **(H)** Tumor volume of mice after treatment. Copyright Kim et al. (2016) Nature publishing group, Zhao et al. (2015) American Chemical Society, and Chen et al. (2015) Elsevier.

Graphene oxide is capable of dynamically interacting with the probe and/or for the transduction of a specific response toward the target molecules. This transduction process is achieved by fluorescence, Raman scattering and electrochemical reaction. On the basis of this, GO are broadly used as biosensors (Kim et al., 2017; Suvarnaphaet and Pechprasarn, 2017), and we discuss here the most recent works on the progress of GO-based nanoarchitecture in biosensing applications. Graphene nanomaterials have been extensively used for the selective electrochemical sensing of single- and double-stranded DNA (Liu et al., 2012; Tang et al., 2015). The high sensitivity could be attributed to the excellent electrochemical properties of graphene, the strong ionic interaction between the negatively charged –COOH groups and the positively charged nucleobases, and the robust π–π stacking between the nucleobases and honeycomb carbon framework. The Rahigi group developed reduced graphene nanowire (RGNW) biosensors for electrochemical detection of the four bases of DNA (guanine, tyrosine, adenine and cytosine) by checking oxidation signals of the discrete nucleotide bases (Akhavan et al., 2012). The RGNW exhibited tremendous stability, with only 15% variation in the oxidation signals upon an increase in differential pulse voltammetry (DPV) up to 100 scans. Recently, Zhang and co-workers designed carboxyl (-COOH) functionalized GO and polyaniline (PANI)-modified GO. They successfully detected DNA via DPV with ranges between 1 × 10^-6^ and 1 × 10^-14^ (Cheng et al., 2017). Johnson and co-workers designed a label-free DNA biosensor based on graphene field effect transistors (GEFTs) functionalized with single-stranded probe DNA. This highly sensitive biosensor offered a broad analytical range with a detection limit of 1 fM for 60-mer DNA oligonucleotides (Ping et al., 2016). By the same group, a device based on gold nanoparticle-decorated GEFTs (Au NP-Gr-FETs) was fabricated by the physical vapor deposition method. Thiol-functionalized Au NP-Gr-FETs were able to detect DNA with a detection limit of 1 nM and exhibited high specificity against no complementary DNA (Gao et al., 2016). A single-layer graphene (SLG)-based FET biosensor was able to detect a very low concentration of DNA (10 fM) (Zheng et al., 2015). Kim et al. (2016) developed a graphene surface modified vertically aligned silicon nanowire for detecting oligonucleotides with sensitivity and selectivity. They first decorated oligonucleotides on the surface of Si nanowire arrays and followed by hybridization to the probe, resulting in an increase in the biosensor (Figure 3A). It was observed that the current of the biosensor was increased from 19 to 120% with an increase in concentration of DNA from 0.1 to 500 nM (Figure 3B; Kim et al., 2016). Park et al. (2014) evaluated the adsorption and desorption mechanism of single- and double-stranded DNA on GO. They observed that ssDNAs were preferentially adsorbed on GO whereas dsDNA exhibited lower affinity. Alternatively, recently it was studied that adsorption of DNA on GO is length-dependent (Huang and Liu, 2018). Prabowoa et al. (2016) introduced a novel idea for the detection of *Mycobacterium tuberculosis* DNA hybridization using graphene deposited on a SPR-sensing chip. The use of GO-based nanomaterials for glucose sensing is now growing prosperously (Cheng et al., 2017; Kumar et al., 2017). A device based on graphene gated electrodes with glucose oxidase exhibited superior selectivity and enhanced glucose sensitivity with a detection limit of 0.5 mM (Zhang et al., 2015). The Jun group fabricated reduced graphene oxide (RGO) with phenyl butyric acid (PBA), which could be used as a linker to bind glucose. The well-modulated RGO-based radio frequency (RF) sensor device was capable of detecting glucose levels in the range between 1 and 4 mM (Park et al., 2016). The Chen Group prepared a highly stable and reusable graphene-bismuth composite device, which was capable of detecting glucose in a wide linear range of 1–12 mM with a high sensitivity of 2.243 μAmM^-1^cm^-2^ and with a low detection limit of 0.35 mM (Mani et al., 2015). Carbon modified graphene/fullerene C60 composite was fruitfully designed to detect glucose in the range of 0.1–12.5 mM. The device showed a limit of detection (LOD) of 35 μM, with high sensitivity of 55.97 μAmM^-1^cm^-2^ (Thirumalraj et al., 2015). Ponpandian’s group successfully developed hydroxyapatite 1-D nanorods on a graphene nanosheet modified with glassy carbon electrode. The device exhibited an excellent sensing property in a wide range of 0.1–11.5 mM with a LOD of 0.03 mM and greater sensitivity of 16.9 μAmM^-1^cm^-2^ (Bharath et al., 2015).

### Graphene Oxide for Drug Delivery

Utilizing the extremely large surface area and available π electrons, graphene is suitable as a drug carrier. Wang et al. (2012) loaded a high amount of doxorubicin (DOX) on phospholipid monolayer coated graphene and subsequently observed the sustained release of DOX to a greater extent at an acidic pH compared to a basic pH (Liu et al., 2012). DOX could be loaded on a graphene sheet via physisorption followed by surface modification by PEG-NH_2_ in order to enhance stability and compatibility in a biological medium (Zhang et al., 2013). Nandi and co-workers were able to load both a hydrophilic drug (DOX) and a hydrophobic drug (indomethacin) successfully on poly-N-isopropyl acrylamide (PNIPAM) grafted GO (GPNM) via π–π interaction, H-bonding and hydrophobic interaction (Kundu et al., 2015). They grafted PNIPAM covalently with GO through the free radical polymerization process (FRPP). The controlled release of DOX was favorable in an acidic pH due to the enhancement of hydrophilicity, higher solubility to DOX and a minimization of the hydrogen bonding interaction between DOX and the GPNM surface. Xu et al. (2014) loaded paclitaxel (PTX) onto GO-PEG via π–π stacking and hydrophobic interactions and the loading capacity was calculated to be 11.2 wt%. Zhao et al. (2015) designed well-defined polymethylmethacrylic acid (PMMA)-coated polyethylene glycol (PEG) modified graphene oxide nanoparticles (GON), which were highly dispersed in PBS solution, and acted as an efficient drug delivery system (Figure 3C). PMMA brushes capably reduce the impulsive release of DOX in the stimulated normal tissues and accelerates DOX release in the tumor tissues in response to a reducing agent, glutathione (GSH) (10 μM) (Figure 3D). Furthermore, strong fluorescence of DOX (green) indicated a persistent release of DOX from DOX-loaded PEGylated alginate (ALG-PEG) grafted GON and its internalization (Figure 3E; Zhao et al., 2014). The Tan group designed DOX-loaded GO followed by modification with hyaluronic acid (HA), which was used as a targeting agent and to enhance the stability of the HA-GO-DOX nanohybrid (Song et al., 2014). Encouraged by the high loading of DOX on GO, recently Mahdavi et al. (2016) have fruitfully carried out a simulation study on DOX loading and releasing in GO at different pH points. In doxorubicin (DOX)-loaded p-aminobenzoic acid polyethyleneimine (PEI), biotin, b-Cyclodextrin (b-CD) conjugated graphene oxide (rGO) nanosystem, the PEI and biotin were used to enhance the stability and targeting efficacy, respectively. The b-Cyclodextrin (b-CD) acted as host molecules for accommodating guest molecules, such as water insoluble anticancer drugs (Wei et al., 2014).

### Graphene Oxide for Cancer Therapy

Recently, GO has been considered to be an exciting nanomaterial due to its inherent size- and shape-dependent optical properties, unique physicochemical behavior, extremely large surface to volume ratio and versatile surface properties, which make it ideal nanomaterial for cancer therapy (Kumar et al., 2017; Nejabat et al., 2017). Yu et al. (2017) designed α_v_β6-targeting peptide (HK-peptide) functionalized and photosensitizer (HPPH) coated GO (GO (HPPH)-PEG-HK). The GO (HPPH)-PEG-HK activated dendritic cells and significantly prevented tumor growth and lung metastasis by increasing the infiltration of cytotoxic CD8^+^ T lymphocytes within tumors as evidenced by *in vivo* optical and single-photon emission computed tomography (SPECT)/CT imaging (Yu et al., 2017). The Chen Group fabricated a DOX-loaded RGO-gold nanorods vehicle for combined photothermal therapy and chemotherapy. A large release of DOX was observed due to the NIR photothermal heating effect and acidic nature of the tumor microenvironment (Song et al., 2015). The tight packing of Au NPS on GO led to an enhancement of the absorption peak from 528 to 600 nm. Under laser light (808 nm, 1.0 W/cm^2^), Au (30 nm)-GO (20 nm) showed the maximum temperature increase of 23.2°C (Kang et al., 2017). Cheon et al. (2016) claimed that a DOX-loaded BSA functionalized graphene sheet could be a powerful tool for combining chemo- and photothermal therapy for brain tumors. Regarding the clinical application, the Chen Group fabricated hyaluronic acid-chitosan-g-poly (N-isopropyl acrylamide) (HACPN) grafted DOX-folic acid-GO thermosensitive hydrogel for breast cancer therapy (Fong et al., 2017). Su et al. (2016) designed a novel material consisting of dual chemotherapeutics loaded sponge-like carbon material on graphene nanosheet (graphene nanosponge) supported lipid bilayers (lipo-GNS) modified with tumor targeting protein. The well fabricated ultrasmall lipo-GNS (40 nm) showed a significant accumulation in the tumor site and, therefore, successful suppression of the xenograft tumors in 16 days (Su et al., 2016). Shao et al. (2017) designed a mesoporous silica (MS) coated polydopamine that functionalized RGO followed by modification with hyaluronic acid (HA) and DOX loading. The pH dependent and near infrared-triggered DOX release made the RGO@MS (DOX)-HA an effective chemo-photothermal agent (Shao et al., 2017). Very recent, Dai et al. (2017) designed TiO_2_-MnOx conjugated graphene composite as a smart material for tumor eradication. Our group developed ^131^I labeled PEG functionalized nano RGO for combined radio and photothermal therapy (Figure 3F). Effectual tumor accumulation of ^131^I-RGO-PEG was observed after its intravenous injection as confirmed by gamma imaging (Figure 3G). RGO exhibited strong near-infrared (NIR) absorbance and could induce effective photothermal heating of the tumor under NIR light irradiation. ^131^I was able to emit b rays to kill cancer cells (Figure 3H; Chen et al., 2015). Some more recent studies based on GO nanomaterials for different cancer therapies have been listed in Table 1.

## Graphene Quantum Dots (GQDs) for Biomedical Applications

### Graphene Quantum Dots (GQDs) as Biosensors

**FIGURE 4:**
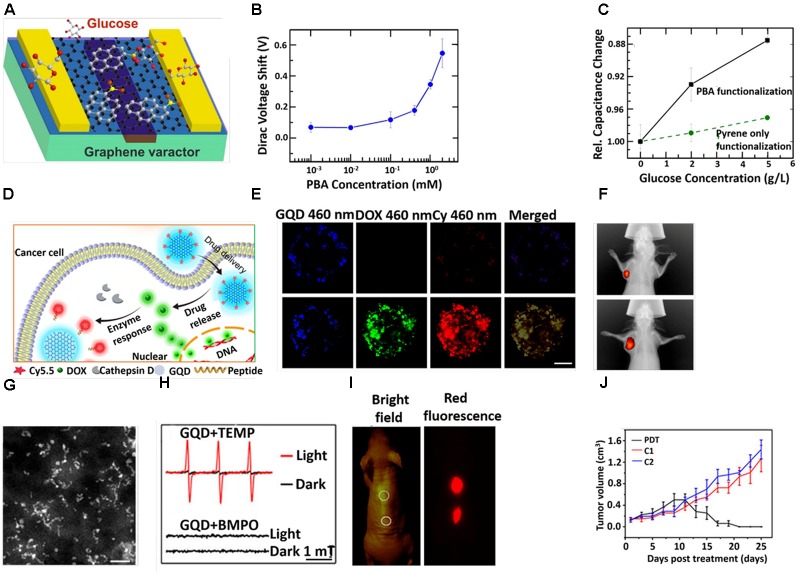
**(A)** Schematic diagram of GQD-based varactor for glucose sensing. **(B)** Dirac voltage shift of graphene varactors with different concentrations of PBA solutions. **(C)** Curves for change in relative capacitance with glucose concentrations. **(D)** Schematic diagram for the drug delivery and release of GQD-based theranostic agent. **(E)** CLSM images of the multicellular tumor spheroids incubation with GQD-P-Cy and DOX@GQD-P-Cy. Scale bar: 100 μm. **(F)** *In vivo* fluorescence images of 4T1 tumor bearing mice after intravenous treatment with DOX@GQD-P-Cy. **(G)** STEM images. Scale bar, 20 nm. **(H)** The electron spin resonance (ESR) signals of ^1^O_2_ (up) and reactive oxygen species (ROS) (down) generated upon irradiation of GQDs for 8 min in the presence of 2,2,6,6-tetramethylpiperidine and 5-tert-butoxycarbonyl-5-methyl-1-pyrroline N-oxide, respectively. **(I)** *In vivo* fluorescence images of GQDs. **(J)** Time dependent tumor growth curves after different treatments. Copyright Ding et al. (2017) and Zhang D.Y. et al. (2017); American Chemical Society and Ge et al. (2014) Nature publishing group.

Recently, GQD-based biosensors have largely been developed for practical applications in clinical analysis and disease diagnosis. On the basis of excellent photoluminescence (PL), electro chemiluminescence (ECL) and electrochemical behaviors of GQD, these have been widely used for detecting bio-macromolecules including DNA, RNA, proteins or glucose molecules with better selectivity and sensitivity (Xie et al., 2016; Kumawat et al., 2017). Qian et al. (2014) developed DNA probe-functionalized reduced GQDs to detect DNA based on the Furrier Resonance Energy Transfer (FRET) fluorescence sensing method. The Qui group successfully designed a Zr^4+^ coordinated phosphorylated peptide-GQD conjugate that was capable to detect casein kinase II (CK2) in the range between 0.1 and 1.0 ml^-1^ with a detection limit of 0.03 ml^-1^ (Wang et al., 2013). Zhang et al. (2017) developed pyrene-1-boronic acid (PBA) functionalized GQD for glucose sensing (Figure 4A). They observed that glucose sensitivity was strongly dependent on the PBA concentration as revealed from the significant shift of Dirac voltage with an increase in the concentration of PBA (Figure 4B). Moreover, the significant enhancement of relative capacitance with an increase in glucose concentration further suggested that the PBA functionalized GQD could be used as a perfect probe for glucose sensing (Figure 4C). The Wei group prepared an electro-chemifluorescent polyvinyl alcohol (PVA)/GQD nanofiber for highly sensitive and selective detection of both H_2_O_2_ and glucose (Zhang et al., 2015). Here, after adsorption of glucose oxidase (GOD) onto the (PVA)/GQD nanofiber, the molecular recognition between GQD and glucose triggered the production of H_2_O_2_, which was detected by fluorescent GQD. The detection of cancer cells in early stage of disease has become a perquisite paradigm. In this regard, Wang et al. (2016) designed Pd NPs decorated N-doped GQD (NGQD) for cancer detection. The NGQD@NC@Pd HNS hybrid material exhibited significant electrochemical reduction of H_2_O_2_. Hence, it was possible to detect various living cancer cells (Xi et al., 2016).

### Graphene Quantum Dots (GQDs) for Drug Delivery

Graphene quantum dots possesses some unique features, such as a single atomic layer with small lateral size and an oxygen-rich surface that renders it suitable for loading drug molecules and enhancing stability in physiological media. In addition, the fluorescent property of GQD makes it an appropriate platform for the traceable delivery of the drug into the cancer cells (Cheng et al., 2015; Pistone et al., 2016; Srivastava et al., 2016). Hence, GQDs have been widely used for drug delivery in various diseases from last decade. The Zhu group loaded DOX on a GQD-embedded zeolite imidazolate framework (ZIF-8), where ZIF-8 was used as an efficient drug carrier. DOX-loaded ZIF-8/GQD nanoparticles effectively showed acidic pH responsive drug release behavior (Tian et al., 2017). Intracellular drug delivery and the real-time monitoring of drug release could be possible from DOX-loaded aptamer/GQD capped fluorescent mesoporous silica nanoparticles. In the adenosine triphosphate (ATP)-rich cytoplasm of the tumor cells, the ATP aptamer caused the release of the GQDs from nanocarriers, resulting in the release of DOX (Zhang et al., 2015). On the basis of the salient physicochemical properties of GQDs, the Wei group developed DOX loaded GQD followed by conjugation with Cy5.5 dye via a cathepsin D-responsive (P) peptide (Ding et al., 2017). The drug-loaded nanoconjugate showed improved tissue penetration and cellular uptake properties, which in turn facilitated superior therapeutic performance both *in vitro* and *in vivo*. The cellular uptake of 4T1 cells and release of DOX were evaluated by confocal laser scanning microscopy (CLSM) (Figure 4E). The GQD-P-Cy treated cells exhibited blue fluorescence, implying promising internalization. The invisible fluorescence signal of Cy5.5 from GQD-P-Cy treated cells indicted its satisfactory biocompatibility. The green fluorescence signal around 565 nm from the DOX@GQD-P-Cy treated cells demonstrated the DOX releasing from GQD. The strong *in vivo* fluorescence signal of DOX from the tumor site signified the great accumulation of DOX inside the tumor (Figure 4F). Nigam et al. (2014) developed a GQD-conjugated gemcitabine-loaded HSA nanoformulation for targeted drug delivery. In this nanosystem, albumin helped to deliver gemcitabine to the tumor cells via the gp60 pathway (Nigam et al., 2014). Pietro and colleagues designed biotin-conjugated DOX-loaded GQD for targeted drug delivery in cancer therapy (Iannazzo et al., 2017). Sui et al. (2016) fabricated a cisplatin-GQD nanoconjugate for enhanced anticancer activity. In this nanoconjugate, GQD helped to improve cellular uptake and then cisplatin assisted to enhance nuclear uptake by interacting with DNA (Sui et al., 2016). Wang et al. (2014) demonstrated that ligand modified DOX-loaded GQD-folic acid nanocarrier improved selective cell labeling, targeted drug delivery and the real-time monitoring of cellular uptake.

### Graphene Quantum Dots (GQDs) for Cancer Therapy

Owing to its outstanding physicochemical property, low toxicity, good hydrophilicity, stable intrinsic fluorescence property and surface functional groups, various kinds of nanomedicines, from chemotherapeutics to radioisotopes, were conceivable for loading and usage for cancer treatments (Iannazzo et al., 2017). The Lee group fabricated hydrophobic anticancer drug, curcumin loaded GQDs for synergistic chemotherapy (Some et al., 2014). Ge et al. (2014) synthesized GQD, which showed tremendous singlet oxygen (^1^O_2_) generation capability and photodynamic therapy (PDT) via *in vivo* therapy. The diameter of GQD was in the range between 2 and 6 nm as revealed from scanning transmission electron microscopy (STEM) (Figure 4G). Their group explored how GQD was able to generate singlet oxygen (^1^O_2_) under irradiation in presence of 2, 2, 6,6-tetramethylpiperidine as observed from ESR peaks (Figure 4H). However, absence of an ESR signal in the presence of 5-tert-butoxycarbonyl-5-methyl-1-pyrroline N-oxide under irradiation indicated that no other ROS was generated. Moreover, no significant diffusion of GQD was at the injection site (Figure 4I). On *in vivo* PDT, a tumor of female BALB/c mice treated with GQD started to diminish after 9 days and after 17 days (Figure 4). Yao et al. (2017) explored that GQD capped magnetic mesoporous silica nanoparticles have the ability to produce heat under an alternating magnetic field (AMF) and/or under NIR irradiation. The material exhibited efficient chemo-photothermal therapy and magnetic hyperthermia as revealed from an *in vitro* study (Yao et al., 2017). The Fan group loaded IR780 on folic acid functionalized GQD for targeted photothermal therapy. Upon irradiation with an 808 nm laser for 5 min, the temperature at the tumor site of the IR780/GQD-FA treated mice increased abruptly to 58.9°C and *in vivo* antitumor study exhibited a clear suppressive effect on tumor growth, and the tumor had almost dissipated by the 15th day (Li et al., 2017). Other studies based on GQDs for different cancer therapies are listed in Table 1.

## Toxicity of Carbon Nanomaterials

**FIGURE 5:**
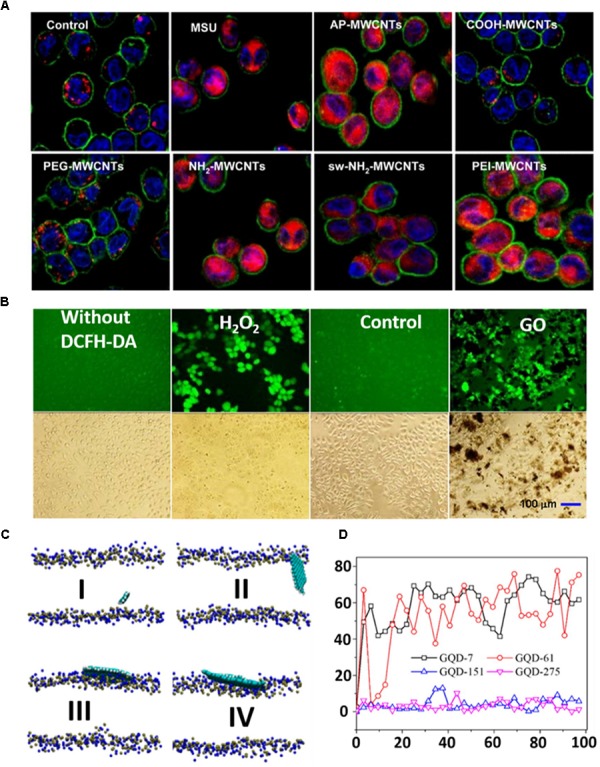
**(A)** Visualization of cathepsin B localization in THP-1 cells exposed to tubes. Lysosomal damage and cathepsin B release were identified by using Magic Red staining. THP-1 cells were seeded into 8-well chamber slides and incubated with f-MWCNTs at 120 μg/ml in complete RPMI 1640 for 3 h. After fixation, cells were stained with Magic Red (ImmunoChemistry Technologies), wheat germ agglutinin-Alexa 488, and Hoechst 33342 dye, followed by visualization under a confocal 1P/FCS inverted microscope. **(B)** ROS production in GLC-82 cells treated with 100 mg/L of GO for 48 h. The positive control was prepared by culturing the cells with RPMI-1640 containing 100 μM of H_2_O_2_ for 1 h prior to the addition of DCFH-DA. The cells without DCFH-DA treatment was taken as a negative control. The control means that cells without exposure to GO were labeled by the DCFH-DA. **(C)** GQDs with different sizes on the membrane after 100 ns MD simulation: (I) GQD7-small size, (II) GQD61-small size, (III) GQD151-large size, and (IV) GQD275-large size. The GQDs are shown by a VDW model with VMD. N atoms (blue) and P atoms (yellow) in the membrane are also shown in the VDW model. **(D)** The angles between different GQDs and the x–y plane of the lipid membrane as a function of simulation time. Copyright Li et al. (2013, 2014) and Liang et al. (2016) American Chemical Society.

Carbon nanomaterials are a novel class of materials that are widely used in biomedical fields including the delivery of therapeutics, biomedical imaging, biosensors, tissue engineering and cancer therapy. However, they still suffer from their toxic effect on biological systems. Until now, various investigations have been carried out on the toxicity of CNT (Liu et al., 2013; Madani et al., 2013; Allegri et al., 2016; Kobayashi et al., 2017). From numerous studies it has been revealed that several factors contribute to the toxicity of CNT. The effect of metal impurities in CNT could have a substantial impact on toxicity (Koyama et al., 2009; Vittorio et al., 2009; Aldieri et al., 2013). The impurities, such as metal ions, were incorporated inside the CNT during synthesis and caused toxicity to the cells. The length of CNT has a great impact on the toxicity of CNT only due to the failure of their cellular internalization (Kostarelos, 2008). Some groups have prepared CNT with different sizes and studied their toxic behavior on cells or DNA (Smart et al., 2006; Raffa et al., 2008). The Donaldson group described that long-term retention of long CNT led to severe inflammation, which caused progressive fibrosis (Murphy et al., 2011). Moreover, the higher diameter with equal average length of CNT exhibits greater toxicity (Kolosnjaj-Tabi et al., 2010). Owing to the difference in size, structure and chemical surface states between SWCNT and MWCNT, they delivered different toxicity effects on cells (Fraczek et al., 2008; DiGiorgio et al., 2011). Moreover, the solubilizing agents played an important role in the toxicity of CNT (Nam et al., 2011; Kim et al., 2012). The individual CNTs tend to bundle in presence of some natural dispersants and led to toxicity. Interestingly, surface functionalization of CNT triggered toxicity in cells. The Jos group found that –COOH functionalized SWCNT induced higher toxicity compared to the non-functionalized SWCNT in the HUVEC cell lines (Praena et al., 2011). On the other hand, Li et al. (2013) demonstrated that strongly cationic functionalized MWCNT has greater potential for lysosomal damaging due to their high cellular uptake and NLRP3 inflammasome activation in comparison to the carboxyl group-functionalized or moderately amine group-functionalized MWCNT, as can be observed by confocal imaging (Figure 5A; Li et al., 2013). Like CNT, graphene has also limitations to biomedical application due to its toxicity. Ou et al. (2016) thoroughly described in their recent review article the toxicity of graphene in different organs. Numerous studies have been conducted on the toxicity of graphene in animals and cells (Shareena et al., 2018). It was stated that several parameters, including concentration, lateral dimension, surface property and functional groups, greatly influence its toxicity in biological systems (Seabra et al., 2014; Alshehri et al., 2016). Li et al. (2014) observed that GO at a concentration of 100 mg/L induced reactive oxygen species (ROS) production in GLC-82 cells upon incubation for 24 h and caused toxicity (Figure 5B). To overcome the toxic effect of GO in various biomedical applications, many research groups have designed GO with various biological molecules. The Zhou group modified a graphene sheet by coating it with blood protein to reduce its toxic effect (Chong et al., 2015). Among different materials of the carbon family, GQDs contain some exciting properties and these have thus been extensively used for biological applications as discussed above. The toxicity of GQDs is different from graphene and GO, thus it is an imperative and serious issue that ought to be addressed. After many investigations, it has been implied that various parameters govern the toxicity of GQDs. It seems that the smaller size of GQDs is an advantage over GO or CNT in terms of toxicity. More importantly, Wang et al. (2016) showed a cell viability mapping curve for various cells under the same conditions and concluded that GQDs with a size below 10 nm possess extremely high cell viability. No doubt, the concentration of nanomaterials is a dominating factor in toxicity. For GQDs, the concentration tolerance of the cells to different GQDs is contradictory. The Shen group showed theoretically that the potential cytotoxicity of GQDs depends on their size and concentration (Liang et al., 2016). They observed that in the 100 ns scale simulation, GQDs with relatively small size could permeate into the POPC membrane (Figure 5C). The permeation of GQDs could affect the thickness of the POPC lipid membrane. At the starting point, angles between GQDs and lipid membrane were 0° in all cases. During simulation, smaller-size GQDs permeated the POPC membrane and created an angle in the range between 45° and 70°. GQDs with larger sizes were only absorbed on the lipid membrane surface and formed an angle in the range of 0° to 10° (Figure 5D). Moreover, it has been observed that the surface functional groups of nanomaterials have a great impact on the toxicity of nanomaterials. The Shang group reported after an investigation that hydroxylated-GQDs have significant toxicity on A549 and H1299 cells (Tian et al., 2016). In contrast, Nurunnabi et al. (2013) claimed that carboxylated GQDs had no acute toxicity on different cancer cells such as KB, MDA-MB231, A549 and the normal cell line such as MDCK. Furthermore, after a long-term *in vivo* study they did not find notable damage to the organs. Regrettably, we have not yet found any article that gives clear information based on the effect of different functional groups in the toxicity of GQD nanomaterials.

## Future Prospective and Conclusion

Over the last two decades, widespread research efforts have been conducted on CBNs as one of the most widely used classes of nanomaterials. Having their inherent mechanical, optical, electrochemical and electrical properties, CBNs have been extensively used in multiple areas. In addition, owing to their versatile surface properties, size and shape over the past decade, CBNs have drawn great attention in biomedical engineering. Interestingly, CBNs are becoming promising materials due to the existence of both inorganic semiconducting properties and organic π–π stacking characteristics. Hence, it could effectively interact with biomolecules and response to the light simultaneously. By taking advantage of such aspects in a single entity, CBN-based nanomaterials could be used for developing biomedical applications in future. Concerning their toxic effect in the biological system, several chemical modification strategies have been developed and successfully used in bio-applications including drug delivery, tissue engineering, detection of biomolecules and cancer therapy. This review article provides some achievements in the use of CBNs for biomedical applications. Moreover, in this paper we also focus on some recently found key features of CBNs and their utilizations for superior bio-applications. However, as CBNs still contain toxicity, more systematic studies are needed to determine the toxicity and pharmacokinetics of CBNs.

## Author Contributions

DM and XT wrote the manuscript. KY and XM revised the manuscript.

## Conflict of Interest Statement

The authors declare that the research was conducted in the absence of any commercial or financial relationships that could be construed as a potential conflict of interest.

## References

[B1] Adeli M. Soleyman R. Beiranvanda Z. Madani F. (2013). Carbon nanotubes in cancer therapy: a more precise look at the role of carbon nanotube-polymer interactions. *Chem. Soc. Rev.* 42 5231–5256. 10.1039/c3cs35431h 23443245

[B2] Akhavan O. Ghaderi E. Rahighi R. (2012). Toward single-DNA electrochemical biosensing by graphene nanowalls. *ACS Nano* 6 2904–2916. 10.1021/nn300261t 22385391

[B3] Aldieri E. Fenoglio I. Cesano F. Gazzano E. Gulino G. Scarano D. (2013). The role of iron impurities in the toxic effects exerted by short multiwalled carbon nanotubes (MWCNT) in murine alveolar macrophages. *J. Toxicol. Environ. Health A* 76 1056–1071. 10.1080/15287394.2013.834855 24188191

[B4] Allegri M. Perivoliotis D. K. Bianchi M. G. Chiu M. Pagliaro A. Koklioti M. A. (2016). Toxicity determinants of multi-walled carbon nanotubes: the relationship between functionalization and agglomeration. *Toxicol. Rep.* 3 230–243. 10.1016/j.toxrep.2016.01.011 28959543 PMC5615827

[B5] Alshehri R. Ilyas A. M. Hasan A. Arnaout A. Ahmed F. Memic A. (2016). Carbon nanotubes in biomedical applications: factors, mechanisms, and remedies of toxicity. *J. Med. Chem.* 59 8149–8167. 10.1021/acs.jmedchem.5b01770 27142556

[B6] Amenta V. Aschberger K. (2015). Carbon nanotubes: potential medical applications and safety concerns. *Adv. Rev.* 7 371–386. 10.1002/wnan.1317 25429905

[B7] Baldo S. Buccheri S. Ballo A. Camarda M. LaMagna A. Castagna M. E. (2016). Carbon nanotube-based sensing devices for human Arginase-1 detection. *Sens. Biosensing Res.* 7 168–173. 10.1016/j.sbsr.2015.11.011

[B8] Banerjee S. S. Todkar K. J. Khutale G. V. Chate G. P. Biradar A. V. Gawande M. B. (2015). Calcium phosphate nanocapsule crowned multiwalled carbon nanotubes for pH triggered intracellular anticancer drug release. *J. Mater. Chem. B* 3 3931–3939. 10.1039/C5TB00534E 32262615

[B9] Bao Z. Liu X. Liu Y. Liu H. Zhao K. (2016). Near-infrared light-responsive inorganic nanomaterials for photothermal therapy. *AJPS* 11 349–364. 10.1016/j.ajps.2015.11.123

[B10] Bharath G. Madhu R. Chen S. M. Veeramani V. Balamurugan A. Mangalaraj D. (2015). Enzymatic electrochemical glucose biosensors by mesoporous 1D hydroxyapatite-on-2D reduced graphene oxide. *J. Mater. Chem. B* 3 1360–1370. 10.1039/C4TB01651C 32264487

[B11] Bhattacharya K. Mukherjee S. P. Gallud A. Burkert S. C. Bistarelli S. Bellucci S. (2016). Biological interactions of carbon-based nanomaterials: from coronation to degradation. *Nanomedicine* 12 333–351. 10.1016/j.nano.2015.11.011 26707820 PMC4789123

[B12] Bisker G. Dong J. Park H. D. Iverson N. M. Ahn J. Nelson J. T. (2016). Protein-targeted corona phase molecular recognition. *Nat. Commun.* 7:10241. 10.1038/ncomms10241 26742890 PMC4729864

[B13] Cha C. Shin S. R. Annabi N. Dokmeci M. R. Khademhosseini A. (2013). Carbon-based nanomaterials: multifunctional materials for biomedical engineering. *ACS Nano* 7 2891–2897. 10.1021/nn401196a 23560817 PMC3648999

[B14] Chen F. Gao W. Qiu X. Zhang H. Liu L. Luo Y. (2018). Graphene quantum dots in biomedical applications: recent advances and future challenges. *Front. Lab. Med.* 1 192–199. 10.1016/j.flm.2017.12.006

[B15] Chen J. Chen S. Zhao X. Kuznetsova L. V. Wong S. S. Ojim I. (2008). Functionalized single-walled carbon nanotubes as rationally designed vehicles for tumor-targeted drug delivery. *J. Am. Chem. Soc.* 130 16778–16785. 10.1021/ja805570f 19554734 PMC2888730

[B16] Chen L. Zhong X. Yi X. Huang M. Ning P. Liu T. (2015). Radionuclide 131I labeled reduced graphene oxide for nuclear imaging guided combined radio- and photothermal therapy of cancer. *Biomaterials* 66 21–28. 10.1016/j.biomaterials.2015.06.043 26188609

[B17] Cheng D. Yang L. Li X. Zhou J. Chen Q. Yan S. (2017). An electrochemical DNA sensing platform using carboxyl functionalized graphene as the electrode modified material. *J. Electrochem. Soc.* 164 H345–H351. 10.1149/2.0951706jes

[B18] Cheng Y. J. Luo G. F. Zhu J. Y. Xu X. D. Zeng X. Cheng D. B. (2015). Enzyme-induced and tumor-targeted drug delivery system based on multifunctional mesoporous silica nanoparticles. *ACS Appl. Mater. Interfaces* 7 9078–9087. 10.1021/acsami.5b00752 25893819

[B19] Cheon Y. A. Bae J. H. Chung B. G. (2016). Reduced graphene oxide nanosheet for chemo-photothermal therapy. *Langmuir* 32 2731–2736. 10.1021/acs.langmuir.6b00315 26930106

[B20] Chong Y. Ge C. Yang Z. Garate J. A. Gu Z. Weber J. K. (2015). Reduced cytotoxicity of graphene nanosheets mediated by blood-protein coating. *ACS Nano* 9 5713–5724. 10.1021/nn5066606 26040772

[B21] Dai C. Zhang S. Liu Z. Wu R. Chen Y. (2017). Two-dimensional graphene augments nanosonosensitized sonocatalytic tumor eradication. *ACS Nano* 11 9467–9480. 10.1021/acsnano.7b05215 28829584

[B22] Das M. Singh R. P. Datir S. R. Jain S. (2013). Correction to “Intranuclear drug delivery and effective in vivo cancer therapy via estradiol-PEG-appended multiwalled carbon nanotubes. *Mol. Pharm.* 10 3404–3416. 10.1021/mp4002409 23905512

[B23] Deng L. Li Q. Rehili S. A. Omar H. Almalik A. Alshamsan A. (2016). Hybrid iron oxide-graphene oxide-polysaccharides microcapsule: a micro-matryoshka for on-demand drug release and antitumor therapy in vivo. *ACS Appl. Mater. Interfaces* 8 6859–6868. 10.1021/acsami.6b00322 26915062

[B24] Dhar S. Liu Z. Thomale J. Dai H. Lippard S. J. (2008). Targeted single-wall carbon nanotube-mediated Pt (IV) prodrug delivery using folate as a homing device. *J. Am. Chem. Soc.* 130 11467–111476. 10.1021/ja803036e 18661990 PMC2536766

[B25] DiGiorgio M. L. DiBucchianico S. Ragnelli A. M. Aimola P. Santucci S. Poma A. (2011). Effects of single and multi-walled carbon nanotubes on macrophages: cyto and genotoxicity and electron microscopy. *Mutat. Res. Genet. Toxicol. Environ. Mutagen.* 722 20–31. 10.1016/j.mrgentox.2011.02.008 21382506

[B26] Ding H. Zhang F. Zhao C. Lv Y. Ma G. Wei W. (2017). Beyond a Carrier: graphene quantum dots as a probe for programmatically monitoring anti-cancer drug delivery, release, and response. *ACS Appl. Mater. Interfaces* 9 27396–27401. 10.1021/acsami.7b08824 28782357

[B27] Dong X. Sun Z. Wang X. Leng X. (2017). An innovative MWCNTs/DOX/TC nanosystem for chemo-photothermal combination therapy of cancer. *Nanomed. Nanotech. Biol. Med.* 13 2271–2280. 10.1016/j.nano.2017.07.002 28712919

[B28] Eatemadi A. Daraee H. Karimkhanloo H. Kouhi M. Zarghami N. Akbarzadeh A. (2014). Carbon nanotubes: properties, synthesis, purification, and medical applications. *Nanoscale Res. Lett.* 9 393–405. 10.1186/1556-276X-9-393 25170330 PMC4141964

[B29] Eldridge B. N. Bernish B. W. Fahrenholtz C. D. Singh R. (2016). Photothermal therapy of glioblastoma multiforme using multiwalled carbon nanotubes optimized for diffusion in extracellular space. *ACS Biomater. Sci. Eng.* 2 963–976. 10.1021/acsbiomaterials.6b00052 27795996 PMC5082186

[B30] Erickson K. Erni R. Lee Z. Alem N. Gannett W. Zettl A. (2010). Determination of the local chemical structure of graphene oxide and reduced graphene oxide. *Adv. Mater.* 22 4467–4472. 10.1002/adma.201000732 20717985

[B31] Eskandari M. Hosseini S. H. Adeli M. Pourjavadi A. (2014). Polymer-functionalized carbon nanotubes in cancer therapy: a review. *Iran Polym. J.* 23 387–403. 10.1007/s13726-014-0228-9 26222588

[B32] Estrada A. C. Silva A. L. D. Trindade T. (2013). Photothermally enhanced drug release by κ-carrageenan hydrogels reinforced with multi-walled carbon nanotubes. *RSC Adv.* 3 10828–10836. 10.1039/C3RA40662H

[B33] Fan Z. Zhou S. Garcia C. Fan L. Zhou J. (2017). pH-Responsive fluorescent graphene quantum dots for fluorescence-guided cancer surgery and diagnosis. *Nanoscale* 9 4928–4933. 10.1039/C7NR00888K 28368056 PMC5501082

[B34] Faria P. C. B. Santos L. I. Coelho J. P. Ribeiro H. B. Pimenta M. A. Ladeira L. O. (2014). Oxidized multiwalled carbon nanotubes as antigen delivery system to promote superior CD8^+^ T cell response and protection against cancer. *Nano Lett.* 14 5458–5470. 10.1021/nl502911a 25115645

[B35] Fiorica C. Mauro N. Pitarresi G. Scialabba C. Palumbo F. S. Giammona G. (2017). Double-network-structured graphene oxide-containing nanogels as photothermal agents for the treatment of colorectal cancer. *Biomacromolecules* 18 1010–1018. 10.1021/acs.biomac.6b01897 28192653

[B36] Fong Y. T. Chen C. H. Chen J. P. (2017). Intratumoral delivery of doxorubicin on folate-conjugated graphene oxide by in-situ forming thermo-sensitive hydrogel for breast cancer therapy. *Nanomaterials* 7:E388. 10.3390/nano7110388 29135959 PMC5707605

[B37] Fraczek A. Menaszek E. Paluszkiewicz C. Blazewicz M. (2008). Comparative in vivo biocompatibility study of single- and multi-wall carbon nanotubes. *Acta Biomater.* 4 1593–1602. 10.1016/j.actbio.2008.05.018 18585111

[B38] Gaitán C. G. Rosas R. R. Morallón E. Amorós D. C. (2017). Effects of the surface chemistry and structure of carbon nanotubes on the coating of glucose oxidase and electrochemical biosensors performance. *RSC Adv.* 7 26867–26878. 10.1039/C7RA02380D

[B39] Gao Z. Kang H. Naylor C. H. Streller F. Ducos P. Serrano M. D. (2016). Scalable production of sensor arrays based on high-mobility hybrid graphene field effect transistors. *ACS Appl. Mater. Interfaces* 8 27546–27552. 10.1021/acsami.6b09238 27676459

[B40] Ge J. Lan M. Zhou B. Liu W. Guo L. Wang H. (2014). A graphene quantum dot photodynamic therapy agent with high singlet oxygen generation. *Nat. Commun.* 5:4596. 10.1038/ncomms5596 25105845 PMC4143951

[B41] Hadsell M. Cao G. Zhang J. Burk L. Schreiber T. (2014). Pilot study for compact microbeam radiation therapy using a carbon nanotube field emission micro-CT scanner. *Med. Phys.* 41:061710. 10.1021/acsbiomaterials.6b00290 24877805 PMC4032446

[B42] Han L. Hao Y. N. Wei X. Chen X. W. Shu Y. Wang J. H. (2017). Hollow copper sulfide nanosphere–doxorubicin/graphene oxide core–shell nanocomposite for photothermo-chemotherapy. *ACS Biomater. Sci. Eng.* 3 3230–3235. 10.1021/acsbiomaterials.7b00643 33445365

[B43] Holzinger M. Goff A. L. Cosnier S. (2014). Nanomaterials for biosensing applications: a review. *Front. Chem.* 2:63 10.3389/fchem.2014.00063 PMC4145256 25221775

[B44] Hong G. Diao S. Antaris A. L. Dai H. (2015). Carbon nanomaterials for biological imaging and nanomedicinal Therapy. *Chem. Rev.* 115 10816–10906. 10.1021/acs.chemrev.5b00008 25997028

[B45] Hou G. Zhang L. Ng V. Wu Z. Schulz M. (2016). Review of recent advances in carbon nanotube biosensors based on field-effect transistors. *Nano Life* 6:1642006. 10.1142/S179398441642006X 24467652

[B46] Huang H. Yuan Q. Shah J. S. Misra R. D. (2011). A new family of folate-decorated and carbon nanotube-mediated drug delivery system: synthesis and drug delivery response. *Adv. Drug Deliv. Rev.* 63 1332–1339. 10.1016/j.addr.2011.04.001 21514336

[B47] Huang Z. Liu J. (2018). Length-dependent diblock DNA with poly-cytosine (Poly-C) as high-affinity anchors on graphene oxide. *Langmuir* 34 1171–1177. 10.1021/acs.langmuir.7b02812 28946748

[B48] Hwang J. Y. Shin U. S. Jang W. C. Hyun J. K. Wall I. B. Kim H. W. (2013). Biofunctionalized carbon nanotubes in neural regeneration: a mini-review. *Nanoscale* 5 487–497. 10.1039/C2NR31581E 23223857

[B49] Hwang Y. S. Park S. H. Lee J. W. (2017). Applications of functionalized carbon nanotubes for the therapy and diagnosis of cancer. *Polymers* 9:13 10.3390/polym9010013 PMC6432390 30970690

[B50] Iannazzo D. Pistone A. Salamò M. Galvagno S. Romeo R. Giofré S. V. (2017). Graphene quantum dots for cancer targeted drug delivery. *Int. J. Pharm.* 518 185–192. 10.1016/j.ijpharm.2016.12.060 28057464

[B51] Jacobs C. B. Peairs M. J. Venton B. J. (2010). Review: carbon nanotube based electrochemical sensors for biomolecules. *Anal. Chim. Acta* 662 105–127. 10.1016/j.aca.2010.01.009 20171310

[B52] Jena P. V. Roxbury D. Galassi T. V. Akkari L. Horoszko C. P. Iaea D. B. (2017). A Carbon nanotube optical reporter maps endolysosomal lipid flux. *ACS Nano* 11 10689–10703. 10.1021/acsnano.7b04743 28898055 PMC5707631

[B53] Kang J. H. Kima H. S. Shin U. S. (2017). Thermo conductive carbon nanotube-framed membranes for skin heat signal-responsive transdermal drug delivery. *Polym. Chem.* 8 3154–3163. 10.1039/c7py00570a

[B54] Kim S. W. Lee Y. K. Lee J. Y. Hong J. H. Khang D. (2017). PEGylated anticancer-carbon nanotubes complex targeting mitochondria of lung cancer cells. *Nanotechnology* 10.1088/1361-6528/aa8c31 [Epub ahead of print]. 29053471

[B55] Kim J. Park S. Y. Kim S. Lee D. H. Kim J. H. Kim J. M. (2016). Precise and selective sensing of DNA-DNA hybridization by graphene/Si-nanowires diode-type biosensors. *Sci. Rep.* 6:31984. 10.1038/srep31984 27534818 PMC4989226

[B56] Kim S. H. Lee J. E. Sharker S. M. Jeong J. H. In I. Park S. Y. (2015). In vitro and in vivo tumor targeted photothermal cancer therapy using functionalized graphene nanoparticles. *Biomacromolecules* 16 3519–3529. 10.1021/acs.biomac.5b00944 26451914

[B57] Kim S. W. Kim T. Kim Y. S. Choi H. S. Lim H. J. Yang S. J. (2012). Surface modifications for the effective dispersion of carbon nanotubes in solvents and polymers. *Carbon* 50 3–33. 10.1016/j.carbon.2011.08.011

[B58] Ko N. R. Nafiujjaman M. Lee J. S. Lim H. N. Lee Y. K. Kwon I. K. (2017). Graphene quantum dot-based theranostic agents for active targeting of breast cancer. *RSC Adv.* 7 11420–11427. 10.1039/c6ra25949a

[B59] Kobayashi N. Izumi H. Morimoto Y. (2017). Review of toxicity studies of carbon nanotubes. *J. Occup. Health* 59 394–407. 10.1539/joh.17-0089-RA 28794394 PMC5635148

[B60] Kolosnjaj-Tabi J. Hartman K. B. Boudjemaa S. Ananta J. S. Morgant G. Szwarc H. (2010). In vivo behavior of large doses of ultrashort and full-length single-walled carbon nanotubes after oral and intraperitoneal administration to Swiss mice. *ACS Nano* 4 1481–1492. 10.1021/nn901573w 20175510

[B61] Kostarelos K. (2008). The long and short of carbon nanotube toxicity. *Nat. Biotechnol.* 26 774–776. 10.1038/nbt0708-774 18612299

[B62] Koyama S. Kim Y. A. Hayashi T. Takeuchi K. Fujii C. Kuroiwa N. (2009). In vivo immunological toxicity in mice of carbon nanotubes with impurities. *Carbon* 47 1365–1372. 10.1016/j.carbon.2009.01.028

[B63] Kumar S. Amala G. Gowtham S. M. (2017). Graphene based sensors in the detection of glucose in saliva – a promising emerging modality to diagnose diabetes mellitus. *RSC Adv.* 7 36949–36976. 10.1039/C6AY01023G 27306706

[B64] Kumar S. Ahlawat W. Kumar R. Dilbaghi N. (2015). Graphene, carbon nanotubes, zinc oxide and gold as elite nanomaterials for fabrication of biosensors for healthcare. *Biosens. Bioelectron.* 70 498–503. 10.1016/j.bios.2015 25899923

[B65] Kumawat M. K. Thakur M. Gurung R. B. Srivastava R. (2017). Graphene quantum dots for cell proliferation, nucleus imaging, and photoluminescent sensing applications. *Sci. Rep.* 7:15858. 10.1038/s41598-017-16025-w 29158566 PMC5696518

[B66] Kundu A. Nandi S. Das P. Nandi A. K. (2015). Fluorescent graphene oxide via polymer grafting: an efficient nanocarrier for both hydrophilic and hydrophobic drugs. *ACS Appl. Mater. Interfaces* 7 3512–3523. 10.1021/am507110r 25612470

[B67] Landry M. P. Ando H. Chen A. Y. Cao J. Kottadiel V. I. Chio L. (2017). Single-molecule detection of protein effux from microorganisms using fuorescent single-walled carbon nanotube sensor arrays. *Nat. Nanotechnol.* 12 368–377. 10.1038/nnano.2016.284 28114298 PMC6438169

[B68] Lee P. C. Lin C. Y. Peng C. L. Shieh M. J. (2016). Development of a controlled-release drug delivery system by encapsulating oxaliplatin into SPIO/MWNT nanoparticles for effective colon cancer therapy and magnetic resonance imaging. *Biomater. Sci.* 4 1742–1753. 10.1039/C6BM00444J 27722406

[B69] Li K. Liu W. Ni Y. Li D. Lin D. Su Z. (2017). Technical synthesis and biomedical applications of graphene quantum dots. *J. Mater. Chem. B* 5 4811–4826. 10.1039/C7TB01073G 32263997

[B70] Li S. Zhou S. Li Y. Li X. Zhu J. Fan L. (2017). Exceptionally high payload of the IR780 iodide on folic acid-functionalized graphene quantum dots for targeted photothermal therapy. *ACS Appl. Mater. Interfaces* 9 22332–22341. 10.1021/acsami.7b07267 28643511

[B71] Li R. Wang X. Ji Z. Sun B. Zhang H. Chang C. H. (2013). Surface charge and cellular processing of covalently functionalized multiwall carbon nanotubes determine pulmonary toxicity. *ACS Nano* 7 2352–2368. 10.1021/nn305567s 23414138 PMC4012619

[B72] Li Y. Wu Q. Zhao Y. Bai Y. Chen P. Xia T. (2014). Response of MicroRNAs to *in vitro* treatment with graphene oxide. *ACS Nano* 8 2100–2110. 10.1021/nn4065378 24512264

[B73] Liang C. Diao S. Wang C. Gong H. Teng Liu T. Hong G. (2014). Tumor metastasis inhibition by imaging-guided photothermal therapy with single-walled carbon nanotubes. *Adv. Mater.* 26 5646–5652. 10.1002/adma.201401825 24924258

[B74] Liang L. Kong Z. Kang Z. Wang H. Zhang L. Shen J. W. (2016). Theoretical evaluation on potential cytotoxicity of graphene quantum dots. *ACS Biomater. Sci. Eng.* 2 1983–1991. 10.1021/acsbiomaterials.6b00390 33440534

[B75] Lin G. Mi P. Chu C. Zhang J. Liu G. (2016). Inorganic nanocarriers overcoming multidrug resistance for cancer theranostics. *Adv. Sci.* 3:1600134. 10.1002/advs.201600134 27980988 PMC5102675

[B76] Lin Y. Lu F. Tu Y. Ren Z. (2004). Glucose biosensors based on carbon nanotube nanoelectrode ensembles. *Nano Lett.* 4 191–195. 10.1021/nl0347233

[B77] Liu Y. Dong X. Chen P. (2012). Biological and chemical sensors based on graphene materials. *Chem. Soc. Rev.* 41 2283–2307. 10.1039/C1CS15270J 22143223

[B78] Liu J. Liu K. Feng L. Liu Z. Xu L. (2017). Comparison of nanomedicine-based chemotherapy, photodynamic therapy and photothermal therapy using reduced graphene oxide for the model system. *Biomater. Sci.* 5 331–340. 10.1039/c6bm00526h 27935610

[B79] Liu X. Marangon I. Melinte G. Wilhelm C. Moyon C. M. Pichon B. P. (2014). Design of covalently functionalized carbon nanotubes filled with metal oxide nanoparticles for imaging, therapy, and magnetic manipulation. *ACS Nano* 8 11290–11304. 10.1021/nn5040923 25343751

[B80] Liu Y. Zhao Y. Sun B. Chen C. (2013). Understanding the toxicity of carbon nanotubes. *Acc. Chem. Res.* 46 702–713. 10.1021/ar300028m 22999420

[B81] Liu Z. Chen K. Davis C. Sherlock S. Cao Q. Chen X. (2008). Drug delivery with carbon nanotubes for in vivo cancer treatment. *Cancer Res.* 68 6652–6660. 10.1158/0008-5472.CAN-08-1468 18701489 PMC2562710

[B82] Liu Z. Fan A. C. Rakhra K. Sherlock S. Goodwin A. Chen X. (2009a). Supramolecular stacking of doxorubicin on carbon nanotubes for in vivo cancer therapy. *Angew. Chem. Int. Ed.* 48 7668–7672. 10.1002/anie.200902612 19760685 PMC2824548

[B83] Liu Z. Tabakman S. Welsher K. Dai H. (2009b). Carbon nanotubes in biology and medicine: *in vitro* and *in vivo* detection, imaging and drug delivery. *Nano Res* 2 85–120. 10.1007/s12274-009-9009-8 20174481 PMC2824900

[B84] Liu Z. Sun X. Ratchford N. N. Da H. (2007). Supramolecular chemistry on water-soluble carbon nanotubes for drug loading and delivery. *ACS Nano* 1 50–56. 10.1021/nn700040t 19203129

[B85] Luo Y. Cai X. Li H. Lin Y. Du D. (2016). Hyaluronic acid-modified multifunctional Q-graphene for targeted killing of drug-resistant lung cancer cells. *ACS Appl. Mater. Interfaces* 8 4048–4055. 10.1021/acsami.5b11471 26785717 PMC12998986

[B86] Madani S. Y. Mandel A. Seifalian A. M. (2013). A concise review of carbon nanotube’s toxicology. *Nano Rev.* 4:21521. 10.3402/nano.v4i0.21521 24319547 PMC3851535

[B87] Mahdavi M. Rahmani F. Nouranian S. (2016). Molecular simulation of pH-dependent diffusion, loading, and release of doxorubicin in graphene and graphene oxide drug delivery systems. *J. Mater. Chem. B* 4 7441–7451. 10.1039/c6tb00746e 32263744

[B88] Mani V. Devasenathipathy R. Chen S. M. Subramani B. Govindasamy M. (2015). A novel glucose biosensor at glucose oxidase immobilized graphene and bismuth nanocomposite film modified electrode. *Int. J. Electrochem. Sci.* 10 691–700.

[B89] Mehra N. K. Jain N. K. (2015). Optimization of a Pretargeted strategy for the PET imaging of colorectal carcinoma via the modulation of radioligand pharmacokinetics. *Mol. Pharm.* 12 630–643. 10.1021/acs.molpharmaceut.5b00294 26287993 PMC4696756

[B90] Mejri A. Vardanega D. Tangour B. Gharbi T. Picaud F. (2015). Substrate temperature to control moduli and water uptake in thin films of vapor deposited *N,N*’-Di(1-naphthyl)-*N,N*’-diphenyl-(1,1’-biphenyl)-4,4’-diamine (n.d.). *J. Phys. Chem. B* 119 604–611. 10.1021/acs.jpcb.5b05814 26230183

[B91] Mocan T. Matea C. T. Cojocaru I. Ilie I. Tabaran F. A. Zaharie F. (2014). Photothermal treatment of human pancreatic cancer using PEGylated multi-walled carbon nanotubes induces apoptosis by triggering mitochondrial membrane depolarization mechanism. *J. Cancer* 5 679–688. 10.7150/jca.9481 25258649 PMC4174512

[B92] Monthioux M. Kuznetsov V. L. (2006). Who should be given the credit for the discovery of carbon nanotubes? *Carbon* 44 1621–1623. 10.1016/j.carbon.2006.03.019

[B93] Mostofizadeh A. Li Y. Song B. Huang Y. (2011). Synthesis, properties, and applications of low-dimensional carbon-related nanomaterials. *J. Nanomater.* 2011:685081 10.1155/2011/68508

[B94] Mphuthi N. G. Adekunle A. S. Ebenso E. E. (2016). Electrocatalytic oxidation of epinephrine and norepinephrine at metal oxide doped phthalocyanine/MWCNT composite sensor. *Sci. Rep.* 6:26938. 10.1038/srep26938 27245690 PMC4887908

[B95] Mukhopadhyay S. Maiti D. Saha A. Devi P. S. (2016). Shape transition of TiO2 nanocube to nanospindle embedded on reduced graphene oxide with enhanced photocatalytic activity. *Cryst. Growth Des.* 16 6922–6932. 10.1021/acs.cgd.6b01096

[B96] Murphy F. A. Poland C. A. Duffin R. Al-Jamal K. T. AliBoucetta H. Nunes A. (2011). Length-dependent retention of carbon nanotubes in the pleural space of mice initiates sustained inflammation and progressive fibrosis on the parietal pleura. *Am. J. Pathol.* 178 2587–2600. 10.1016/j.ajpath.2011.02.040 21641383 PMC3124020

[B97] Nafiujjaman M. Revuri V. Park H. K. Kwon K. Cho K. J. Lee Y. K. (2016). Enhanced photodynamic properties of graphene quantum dot conjugated Ce6 nanoparticles for targeted cancer therapy and imaging. *Chem. Lett.* 45 997–999. 10.1246/cl.160388

[B98] Nam C. W. Kang S. J. Kang Y. K. Kwak M. K. (2011). Cell growth inhibition and apoptosis by SDS-solubilized single-walled carbon nanotubes in normal rat kidney epithelial cells. *Arch. Pharmacal Res.* 34 661–669. 10.1007/s12272-011-0417-4 21544732

[B99] Nejabat M. Charbgoo F. Ramezani M. (2017). Graphene as multifunctional delivery platform in cancer therapy. *J. Biomed. Res. Part A* 105 2355–2367. 10.1002/jbm.a.36080 28371194

[B100] Nigam P. Waghmode S. Louis M. Wangnoo S. Chavan P. Sarkar D. (2014). Graphene quantum dots conjugated albumin nanoparticles for targeted drug delivery and imaging of pancreatic cancer. *J. Mater. Chem. B* 2 3190–3195. 10.1039/C4TB00015C 32261580

[B101] Nurunnabi M. Khatun Z. Huh K. M. Park S. Y. Lee D. Y. Cho K. J. (2013). *In Vivo* biodistribution and toxicology of carboxylated graphene quantum dots. *ACS Nano* 7 6858–6867. 10.1021/nn402043c 23829293

[B102] Odom T. W. Huang J. L. Kim P. Lieber M. C. (1998). Atomic structure and electronic properties of single-walled carbon nanotubes. *Nature* 391 62–64. 10.1038/34145

[B103] Ou L. Song B. Liang H. Liu J. Feng X. Deng B. (2016). Toxicity of graphene-family nanoparticles: a general review of the origins and mechanisms. *Part. Fibre Toxicol.* 13:57. 10.1186/s12989-016-0168-y 27799056 PMC5088662

[B104] Panczyk T. Wolski P. Lajtar L. (2016). Coadsorption of doxorubicin and selected dyes on carbon nanotubes. Theoretical investigation of potential application as a pH-controlled drug delivery system. *Langmuir* 32 4719–4728. 10.1021/acs.langmuir.6b00296 27133585

[B105] Park B. Park H. G. Ji J. Cho J. Jun S. C. (2016). A Reduced graphene oxide based radio frequency glucose sensing device using multi-dimensional parameters. *Micromachines* 7:136. 10.3390/mi7080136 30404307 PMC6189738

[B106] Park J. S. Goo N. I. Kim D. E. (2014). Mechanism of DNA adsorption and desorption on graphene oxide. *Langmuir* 30 12587–12595. 10.1021/la503401d 25283243

[B107] Pattnaik S. Swainb K. Lin Z. (2016). Graphene and graphene-based nanocomposites: biomedical applications and biosafety. *J. Mater. Chem. B* 4 7813–7831. 10.1039/C6TB02086K 32263772

[B108] Ping J. Vishnubhotla J. R. Vrudhula A. Johnson A. T. C. (2016). Scalable production of high-sensitivity, label-free DNA biosensors based on back-gated graphene field Effect transistors. *ACS Nano* 10 8700–8704. 10.1021/acsnano.6b04110 27532480 PMC5044806

[B109] Pistone D. Iannazzo S. Ansari C. Milone M. Salamo S. Galvagno S. (2016). Tunable doxorubicin release from polymer-gated multiwalled carbon nanotubes. *Int. J. Pharm.* 515 30–36. 10.1016/j.ijpharm.2016.10.010 27720871

[B110] Prabowoa B. A. Aloma A. Secarioa M. K. Masimd F. C. P. Lai H. C. Hatanakad K. (2016). Graphene-based portable SPR Sensor for the detection of *Mycobacterium tuberculosis* DNA strain. *Procedia Eng.* 168 541–545. 10.1016/j.proeng.2016.11.520

[B111] Praena D. G. Pichardo S. Sánchez E. Grilo A. Cameán A. M. Jos A. (2011). Influence of carboxylic acid functionalization on the cytotoxic effects induced by single wall carbon nanotubes on human endothelial cells (HUVEC). *Toxicol. In Vitro* 25 1883–1888. 10.1016/j.tiv.2011.05.027 21651974

[B112] Qian Z. S. Shan X. Y. Chai L. J. Ma J. J. Chen J. R. Feng H. (2014). A universal fluorescence sensing strategy based on biocompatible graphene quantum dots and graphene oxide for the detection of DNA. *Nanoscale* 6 5671–5674. 10.1039/C3NR06583A 24763693

[B113] Raffa V. Ciofani G. Nitodas S. Karachalios T. D’Alessandro D. Masini M. (2008). Can the properties of carbon nanotubes influence their internalization by living cells? *Carbon* 46 1600–1610. 10.1016/j.carbon.2008.06.053

[B114] Raza K. Kumar D. Kiran C. Kumar M. Guru S. K. Kumar P. (2016). Enhanced antitumor efficacy and reduced toxicity of docetaxel loaded estradiol functionalized stealth polymeric nanoparticles. *Mol. Pharm.* 13 2423–2432. 10.1021/acs.molpharmaceut.5b00281 26375023

[B115] Risi G. Bloise N. Merli D. Cornaglia A. I. Profumo A. Fagnoni M. (2014). *In vitro* study of multiwall carbon nanotubes (MWCNTs) with adsorbed mitoxantrone (MTO) as a drug delivery system to treat breast cancer. *RSC Adv.* 4 18683–18693. 10.1039/C4RA02366H

[B116] Roldo M. Fatouros D. G. (2013). Biomedical applications of carbon nanotubes. *Annu. Rep. Prog. Chem. Sect. C Phys. Chem.* 109 10–35. 10.1039/c3pc90010j

[B117] Ruan J. Wang Y. Li F. Jia R. Zhou G. Shao C. (2018). Graphene quantum dots for radiotherapy. *ACS Appl. Mater. Interfaces* 10 14342–14355. 10.1021/acsami.7b18975 29542912

[B118] Seabra A. B. Paula A. J. de Lima R. Alves O. L. Durán N. (2014). Nanotoxicity of graphene and graphene Oxide. *Chem. Res. Toxicol.* 27 159–168. 10.1021/tx400385x 24422439

[B119] Shao L. Zhang R. Lu J. Zhao C. Deng X. Wu Y. (2017). Mesoporous silica coated polydopamine functionalized reduced graphene oxide for synergistic targeted chemo-photothermal therapy. *ACS Appl. Mater. Interfaces* 9 1226–1236. 10.1021/acsami.6b11209 28004583

[B120] Shareena T. P. McShan D. Dasmahapatra A. K. Tchounwou P. B. (2018). A review on graphene-based nanomaterials in biomedical applications and risks in environment and health. *Nanomicro Lett.* 10:53. 10.1007/s40820-018-0206-4 30079344 PMC6075845

[B121] Shi X. Zheng Y. Wang C. Yue L. Qiao K. Wang G. (2015). Dual stimulus responsive drug release under the interaction of pH value and pulsatile electric field for a bacterial cellulose/sodium alginate/multi-walled carbon nanotube hybrid hydrogel. *RSC Adv.* 5 41820–41829. 10.1039/C5RA04897D

[B122] Singh S. Mehra N. K. Jain N. K. (2016). Development and characterization of the paclitaxel loaded riboflavin and thiamine conjugated carbon nanotubes for cancer treatment. *Pharm. Res.* 33 1769–1781. 10.1007/s11095-016-1916-2 27091032

[B123] Smart S. K. Cassady A. I. Lu G. Q. Martin D. J. (2006). The biocompatibility of carbon nanotubes. *Carbon* 44 1034–1047. 10.1016/j.carbon.2005.10.011

[B124] Sobhani Z. Behnam M. A. Emami F. Dehghanian A. Jamhiri I. (2017). Photothermal therapy of melanoma tumor using multiwalled carbon nanotubes. *Int. J. Nanomedicine* 12 4509–4517. 10.2147/IJN.S134661 28684911 PMC5484561

[B125] Some S. Gwon A. R. Hwang F. Bahn G. H. Yoon Y. Kim Y. (2014). Cancer therapy using ultrahigh hydrophobic drug-loaded graphene derivatives. *Sci. Rep.* 4:6314. 10.1038/srep06314 25204358 PMC4159635

[B126] Song E. Han W. Li C. Cheng D. Li L. Liu L. (2014). Hyaluronic acid-decorated graphene oxide nanohybrids as nanocarriers for targeted and pH-responsive anticancer drug delivery. *ACS Appl. Mater. Interfaces* 6 11882–11890. 10.1021/am502423r 25000539

[B127] Song J. Wang F. Yang X. Ning B. Harp M. G. Culp S. H. (2016). Gold nanoparticle coated carbon nanotube ring with enhanced Raman scattering and photothermal conversion Property for theranostic applications. *J. Am. Chem. Soc.* 138 7005–7015. 10.1021/jacs.5b13475 27193381 PMC5227596

[B128] Song L. Shi J. Lu J. Lu C. (2015). Structure observation of graphene quantum dots by single-layered formation in layered confinement space. *Chem. Sci.* 6 4846–4850. 10.1039/C5SC01416F 28717489 PMC5502401

[B129] Srivastava A. Yadav T. Sharma S. Nayak A. Kumari A. Mishra N. (2016). Polymers in drug delivery. *J. Biosci. Med.* 4 69–84. 10.4236/jbm.2016.41009

[B130] Su S. Wang J. Vargas E. Wei J. Zaguilaìn R. M. Sennoune S. R. (2016). Porphyrin immobilized nanographene oxide for enhanced and targeted photothermal therapy of brain cancer. *ACS Biomater. Sci. Eng.* 2 1357–1366. 10.1021/nl100996u 33434989

[B131] Su Y. Hu Y. Wang Y. Xu X. Yuan Y. Li Y. (2017). A precision-guided MWNT mediated reawakening the sunk synergy in RAS for anti-angiogenesis lung cancer therapy. *Biomaterials* 139 75–90. 10.1016/j.biomaterials.2017.05.046 28595131

[B132] Sui X. Luo C. Wang C. Zhang F. Zhang J. Guo S. (2016). Graphene quantum dots enhance anticancer activity of cisplatin via increasing its cellular and nuclear uptake. *Nanomedicine* 12 1997–2006. 10.1016/j.nano.2016.03.010 27085903

[B133] Sun D. Yan X. Lang J. Xue Q. (2013). High performance supercapacitor electrode based on graphene paper via flame-induced reduction of graphene oxide paper. *J. Power Sources* 222 52–58. 10.1016/j.jpowsour.2012.08.059

[B134] Suvarnaphaet P. Pechprasarn S. (2017). Graphene-based materials for biosensors: a review. *Sensors* 17:E2161. 10.3390/s17102161 28934118 PMC5677231

[B135] Tang L. Wang Y. Li J. (2015). The graphene/nucleic acid nanobiointerface. *Chem. Soc. Rev.* 44 6954–6980. 10.1039/C4CS00519H 26144837

[B136] Thakur M. Kumawat M. K. Srivastava R. (2017). Multifunctional graphene quantum dots for combined photothermal and photodynamic therapy coupled with cancer cell tracking applications. *RSC Adv.* 7 5251–5261. 10.1039/C6RA25976F

[B137] Thirumalraj B. Palanisamy S. Chen S. M. Yang C. Y. Periakaruppan P. Lou B. S. (2015). Direct electrochemistry of glucose oxidase and sensing of glucose at a glassy carbon electrode modified with a reduced graphene oxide/fullerene-C60 composite. *RSC Adv.* 5 77651–77657. 10.1039/C5RA12018G

[B138] Tian X. Xiao B. B. Wu A. Yu L. Zhou J. Wang Y. (2016). Hydroxylated-graphene quantum dots induce cells senescence in both p53-dependent and -independent manner. *Toxicol. Res.* 5 1639–1648. 10.1039/c6tx00209a 30090463 PMC6061981

[B139] Tian Z. Yao X. Ma K. Niu X. Grothe J. Xu Q. (2017). Metal–organic framework/graphene quantum dot nanoparticles used for synergistic chemo- and photothermal therapy. *ACS Omega* 2 1249–1258. 10.1021/acsomega.6b00385 30023630 PMC6044744

[B140] Tîlmaciu C. M. Morris M. C. (2015). Carbon nanotube biosensor. *Front. Chem.* 3:59 10.3389/fchem.2015.00059 PMC4621484 26579509

[B141] Tiwari J. N. Vij V. Kemp K. C. Kim K. S. (2015). Engineered carbon-nanomaterial-based electrochemical sensors for biomolecules. *ACS Nano* 10 46–80. 10.1021/acsnano.5b05690 26579616

[B142] Ulissi Z. W. Sen F. Gong X. Sen S. Iverson N. Boghossian A. A. (2014). Spatiotemporal intracellular nitric oxide signaling captured using internalized, near-infrared fluorescent carbon nanotube nanosensors. *Nano Lett.* 14 4887–4894. 10.1021/nl502338y 25029087 PMC4134139

[B143] Vittorio O. Raffa V. Cuschieri A. (2009). Influence of purity and surface oxidation on cytotoxicity of multiwalled carbon nanotubes with human neuroblastoma cells. *Nanomedicine* 5 424–431. 10.1016/j.nano.2009.02.006 19341817

[B144] Wang J. Cao S. Ding Y. Ma F. Lu W. Sun M. (2016). Theoretical investigations of optical origins of fluorescent graphene quantum dots. *Sci. Rep.* 6:24850. 10.1038/srep24850 27094439 PMC4837401

[B145] Wang L. Wang X. Bhirde A. Cao J. Zeng Y. Huang X. (2014). Carbon-dot-based two-photon visible nanocarriers for safe and highly efficient delivery of siRNA and DNA. *Adv. Healthc. Mater.* 3 1203–1209. 10.1002/adhm.201300611 24692418 PMC4134771

[B146] Wang X. Wang C. Cheng L. Lee S. T. Liu Z. (2012). Noble metal coated single-walled carbon nanotubes for applications in surface enhanced Raman scattering imaging and photothermal therapy. *J. Am. Chem. Soc.* 134 7414–7422. 10.1021/ja300140c 22486413

[B147] Wang Y. Wei H. Lu Y. Wei S. Wujcik E. K. Guo Z. (2015). Multifunctional carbon nanostructures for advanced energy storage applications. *Nanomater* 5 755–777. 10.3390/nano5020755 28347034 PMC5312914

[B148] Wang Y. Zhang L. Liang R. P. Bai J. M. Qiu J. D. (2013). Using graphene quantum dots as photoluminescent probes for protein kinase sensing. *Anal. Chem.* 85 9148–9155. 10.1021/ac401807b 24004085

[B149] Wei G. Dong R. Wang D. Feng L. Dong S. Song A. (2014). Functional materials from the covalent modification of reduced graphene oxide and β-cyclodextrin as a drug delivery carrier. *New J. Chem.* 38 140–145. 10.1039/C3NJ00690E

[B150] Wu H. C. Chang X. Liu L. Zhao F. Zhao Y. (2010). Chemistry of carbon nanotubes in biomedical applications. *J. Mater. Chem.* 20 1036–1052. 10.1039/B911099M

[B151] Xi J. Xie C. Zhang Y. Wang L. Xiao J. Duan X. (2016). Ultrafine Pd nanoparticles encapsulated in microporous Co3O4 hollow nanospheres for in situ molecular detection of living cells. *ACS Appl. Mater. Interfaces* 8 22563–22573. 10.1021/acsami.5b00600 25705983

[B152] Xie R. Wang Z. Zhou W. Liu Y. Fan L. Li Y. (2016). Graphene quantum dots as smart probes for biosensing. *Anal. Methods* 8 4001–4016. 10.1039/C6AY00289G

[B153] Xu H. Liu M. Lan M. Yuan H. Yu W. Tian J. (2016). Mussel-inspired PEGylated carbon nanotubes: biocompatibility evaluation and drug delivery applications. *Toxicol. Res.* 5 1371–1379. 10.1039/c6tx00094k 30090441 PMC6062158

[B154] Xu Z. Wang S. Li Y. Wang M. Shi P. Huang X. (2014). Covalent functionalization of graphene oxide with biocompatible poly (ethylene glycol) for delivery of paclitaxel. *ACS Appl. Mater. Interfaces* 6 17268–17276. 10.1021/am505308f 25216036

[B155] Xuan C. Thuy N. Luyen T. Huyen T. Tuan M. (2017). Carbon nanotube field-effect transistor for DNA sensing. *J. Electron. Mater.* 46 3507–3511. 10.1007/s11664-016-5238-2

[B156] Yang J. Su H. Sun W. Cai J. Liu S. Chai Y. (2018). Dual chemodrug-loaded single-walled carbon nanohorns for multimodal imaging-guided chemo-photothermal therapy of tumors and lung metastases. *Theranostics* 8 1966–1984. 10.7150/thno.23848 29556368 PMC5858512

[B157] Yang K. Feng L. Shi X. Liu Z. (2013). Nano-graphene in biomedicine: theranostic applications. *Chem. Soc. Rev.* 42 530–547. 10.1039/C2CS35342C 23059655

[B158] Yang K. Zhang S. Zhang G. Sun X. Lee S. T. Liu Z. (2010). Graphene in mice: ultrahigh in vivo tumor uptake and efficient photothermal therapy. *Nano Lett.* 10 3318–3323. 10.1021/nl100996u 20684528

[B159] Yang N. Chen X. Ren T. Zhang P. Yang D. (2015). Carbon nanotube based biosensor. *Sens. Actuator B* 207 690–715. 10.1016/j.snb.2014.10.040

[B160] Yang Y. Liu Y. Cheng C. Shi H. Yang H. Yuan H. (2017). Rational design of GO-modified Fe3O4/SiO2 nanoparticles with combined Rhenium-188 and gambogic acid for magnetic target therapy. *ACS Appl. Mater. Interfaces* 9 28195–28208. 10.1021/acsami.7b07589 28793762

[B161] Yao X. Niu X. Ma K. Huang P. Grothe J. Kaskel S. (2017). Graphene quantum dots-capped magnetic mesoporous silica nanoparticles as a multifunctional platform for controlled drug delivery, magnetic hyperthermia, and photothermal therapy. *Small* 13:1602225. 10.1002/smll.201602225 27735129

[B162] Yu X. Gao D. Gao L. Lai J. Zhang C. Zhao Y. (2017). Inhibiting metastasis and preventing tumor relapse by triggering host immunity with tumor-targeted photodynamic therapy using photosensitizer-loaded functional nanographenes. *ACS Nano* 11 10147–10158. 10.1021/acsnano.7b04736 28901740

[B163] Zhang D. Y. Zheng Y. Tan C. P. Sun J. H. Zhang W. Ji L. N. (2017). Graphene oxide decorated with Ru(II)–polyethylene glycol complex for lysosome-targeted imaging and photodynamic/photothermal therapy. *ACS Appl. Mater. Interfaces* 9 6761–6771. 10.1021/acsami.6b13808 28150943

[B164] Zhang M. Liao C. Mak C. H. You P. Mak C. L. Yan F. (2015). Highly sensitive glucose sensors based on enzyme-modified whole-graphene solution-gated transistors. *Sci. Rep.* 5:8311. 10.1038/srep08311 25655666 PMC4319171

[B165] Zhang H. Grüner G. Zhao Y. (2013). Recent advancements of graphene in biomedicine. *J. Mater. Chem. B* 1 2542–2567. 10.1039/C3TB20405G 32260943

[B166] Zhang L. Yuan H. Inscoe C. Chtcheprov P. Hadsell M. Lee Y. (2014). Nanotube x-ray for cancer therapy: a compact microbeam radiation therapy system for brain tumor treatment. *Expert Rev. Anticancer Ther.* 14 1411–1418. 10.1118/1.4873683 25417729 PMC4260778

[B167] Zhang P. Zhao X. Ji Y. Ouyang Z. Wen X. Li J. (2015). Electrospinning graphene quantum dots into a nanofibrous membrane for dual-purpose fluorescent and electrochemical biosensors. *J. Mater. Chem. B* 3 2487–2496. 10.1039/C4TB02092H 32262123

[B168] Zhao Q. Gan Z. Zhuang Q. (2002). Electrochemical sensors based on carbon nanotubes. *Electroanalysis* 14 1609–1613. 10.1002/elan.200290000

[B169] Zhao X. Liu L. Li X. Zeng J. Jia X. Liu P. (2014). Biocompatible graphene oxide nanoparticle-based drug delivery platform for tumor microenvironment-responsive triggered release of doxorubicin. *Langmuir* 30 10419–10429. 10.1021/la502952f 25109617

[B170] Zhao X. Yang L. Li X. Jia X. Liu L. Zeng J. (2015). Functionalized graphene oxide nanoparticles for cancer cell specific delivery of antitumor drug. *Bioconjug. Chem.* 26 128–136. 10.1021/bc5005137 25525819

[B171] Zheng X. T. Ananthanarayanan A. Luo K. Q. Chen P. (2015). Glowing graphene quantum dots and carbon dots: properties, syntheses, and biological applications. *Small* 11 1620–1636. 10.1002/smll.201402648 25521301

[B172] Zhou Q. Zheng J. Qing Z. Zheng M. Yang J. Yang S. (2016). Detection of circulating tumor DNA in human blood via DNA-mediated surface-enhanced Raman spectroscopy of single-walled carbon nanotubes. *Anal. Chem.* 88 4759–4765. 10.1021/acs.analchem.6b00108 27028517

[B173] Zhu L. Deng C. Chen P. Dong X. Su Y. H. Yuan Y. (2014). Glucose oxidase biosensors based on carbon nanotube non-woven fabrics. *Carbon* 67 795–796. 10.1016/j.carbon.2013.10.046

[B174] Zribi B. Roy E. Pallandre A. Chebil S. Koubaa M. Mejri N. (2016). A microfluidic electrochemical biosensor based on multiwall carbon nanotube/ferrocene for genomic DNA detection of *Mycobacterium tuberculosis* in clinical isolates. *Biomicrofluidics* 2:014115. 10.1063/1.4940887 26865908 PMC4744232

